# Ficolin 3 promotes ferroptosis in HCC by downregulating IR/SREBP axis-mediated MUFA synthesis

**DOI:** 10.1186/s13046-024-03047-2

**Published:** 2024-05-03

**Authors:** Yanmei Yuan, Junting Xu, Quanxin Jiang, Chuanxin Yang, Ning Wang, Xiaolong Liu, Hai-long Piao, Sijia Lu, Xianjing Zhang, Liu Han, Zhiyan Liu, Jiabin Cai, Fang Liu, Suzhen Chen, Junli Liu

**Affiliations:** 1 Shanghai Diabetes Institute, Department of Endocrinology and Metabolism, Shanghai Key Laboratory of Diabetes Mellitus, Shanghai Sixth People’s Hospital Affiliated to Shanghai Jiao Tong University School of Medicine, Shanghai, 200233 China; 2 Dalian Institute of Chemical Physics, Chinese Academy of Sciences, Dalian, 116023 China; 3 Department of Pathology, Shanghai Sixth People’s Hospital Affiliated to Shanghai Jiao Tong University School of Medicine, Shanghai, 200233 China; 4 Department of Liver Surgery and Transplantation, Liver Cancer Institute, Zhongshan Hospital, Fudan University; Key Laboratory of Carcinogenesis and Cancer Invasion, Zhongshan Hospital, Shanghai, 200032 China; 5 Department of Endocrinology and Metabolism, Shanghai General Hospital, Shanghai Jiao Tong University School of Medicine, Shanghai, China

**Keywords:** Ficolin 3, Insulin receptor, Ferroptosis, MUFA, De novo lipogenesis

## Abstract

**Background:**

Targeting ferroptosis has been identified as a promising approach for the development of cancer therapies. Monounsaturated fatty acid (MUFA) is a type of lipid that plays a crucial role in inhibiting ferroptosis. Ficolin 3 (FCN3) is a component of the complement system, serving as a recognition molecule against pathogens in the lectin pathway. Recent studies have reported that FCN3 demonstrates inhibitory effects on the progression of certain tumors. However, whether FCN3 can modulate lipid metabolism and ferroptosis remains largely unknown.

**Methods:**

Cell viability, BODIPY-C11 staining, and MDA assay were carried out to detect ferroptosis. Primary hepatocellular carcinoma (HCC) and xenograft models were utilized to investigate the effect of FCN3 on the development of HCC in vivo. A metabonomic analysis was conducted to assess alterations in intracellular and HCC intrahepatic lipid levels.

**Results:**

Our study elucidates a substantial decrease in the expression of FCN3, a component of the complement system, leads to MUFA accumulation in human HCC specimens and thereby significantly promotes ferroptosis resistance. Overexpression of FCN3 efficiently sensitizes HCC cells to ferroptosis, resulting in the inhibition of the oncogenesis and progression of both primary HCC and subcutaneous HCC xenograft. Mechanistically, FCN3 directly binds to the insulin receptor β (IR-β) and its pro-form (pro-IR), inhibiting pro-IR cleavage and IR-β phosphorylation, ultimately resulting in IR-β inactivation. This inactivation of IR-β suppresses the expression of sterol regulatory element binding protein-1c (SREBP1c), which subsequently suppresses the transcription of genes related to de novo lipogenesis (DNL) and lipid desaturation, and consequently downregulates intracellular MUFA levels.

**Conclusions:**

These findings uncover a novel regulatory mechanism by which FCN3 enhances the sensitivity of HCC cells to ferroptosis, indicating that targeting FCN3-induced ferroptosis is a promising strategy for HCC treatment.

**Supplementary Information:**

The online version contains supplementary material available at 10.1186/s13046-024-03047-2.

## Background

Targeting ferroptosis has been identified as a promising approach for the development of cancer therapies [1, 2]. Cancer cells require higher levels of iron and lipid metabolism for growth than normal cells, which theoretically renders them more susceptible to ferroptosis [3]. However, recent studies have revealed that cancer cells, including HCC cells, frequently exhibit ferroptosis resistance [4]. Therefore, it is crucial to thoroughly investigate the underlying mechanisms by which cancer cells evade ferroptosis.

The initiation and execution of ferroptosis are inextricably linked to lipid metabolism, including the metabolism of polyunsaturated fatty acid (PUFA) and MUFA [5, 6]. Specifically, PUFA-containing phospholipids (PUFA-PLs) are vulnerable to free radical-initiated oxidation, and PUFA-PLs peroxidation-induced cell membrane breakage is a key feature of ferroptosis [7]. Notably, some cancers evade ferroptosis through GPX4-dependent downregulation of polyunsaturated ether phospholipids (PUFA-ePLs) [8]. Conversely, MUFA inhibits ferroptosis by blocking the accumulation of lipid reactive oxygen species (ROS) in the plasma membrane and reducing the level of PUFA incorporation into phospholipids [5]. Moreover, exogenous MUFA can promote a ferroptosis-resistant cell state [9]. However, the elusive question of whether cancer cells can modulate MUFA metabolism to develop ferroptosis resistance and the underlying molecular mechanism have not been thoroughly investigated.

Metabolic reprogramming is a long-established hallmark of malignancy. HCC exhibits significant alterations in lipid metabolism, characterized by increased lipid accumulation via lipogenesis [10, 11]. At the molecular level, key lipogenic enzymes, such as acetyl-CoA carboxylase (ACC) and fatty acid synthase (FASN), are upregulated in HCC and other cancers, enhancing fatty acid synthesis and promoting tumor cell survival [12–15]. Moreover, genetic ablation of FASN suppresses fatty acid biosynthesis and inhibits AKT-driven hepatocarcinogenesis [16]. Furthermore, overexpression of stearoyl-CoA desaturase 1 (SCD1), a key desaturase in the synthesis of MUFA, promotes HCC tumor growth and empowers cancer cells with resistance to sorafenib treatment [17]. In line with this, inhibition of SCD1 significantly potentiates the antitumor effect of ferroptosis inducers in ovarian cancer [18].

The complement system, a constitute of the immune system, includes numerous serum proteins and their receptors. Over the past few decades, incremental research has elucidated the crucial role of complement system members in cancer progression [19]. C5a/C5aR1 signaling forms a stabilization complex, regulating β-catenin stability and activation in colonic epithelial cells to promote colorectal tumorigenesis [20]. FCNs, being major components of the complement system, have been reported to suppress cancer progression in different cancers. FCN2 inhibits epithelial-mesenchymal transition-induced metastasis of hepatocellular carcinoma via TGF-β/Smad signaling [21]. FCN3 suppresses the progression in lung and liver carcinoma by inhibiting apoptosis [22, 23]. However, the involvement of complement members, including FCNs, in modulating metabolic reprogramming and ferroptosis in HCC cells remains largely unknown.

In this study, we revealed that FCN3 promoted ferroptosis within HCC by downregulating intracellular MUFA levels. Moreover, we demonstrated that FCN3 inhibited the IR/SREBP1c signaling axis, thereby suppressing MUFA synthesis. We also observed a decrease in FCN3 expression in both human HCC specimens and cancer cells, accompanied by a negative regulation of MUFA abundance by FCN3 in HCC.

## Methods

### Mouse models

Six-week-old male athymic mice (Slaccas) were subcutaneously injected with nsFCN3-overexpressed and control MHCC97-H cells at 7.5 × 10^5^ cells per dot in Matrigel (354234, Corning). After the tumors grew out, the size of the tumors was measured every two days with digital Vernier calipers and calculated as mm^3^ = 0.5 × length × width^2^. After 36 days of injection, the tumors were harvested, followed by weighing and photographing. FCN3 allele knock-in mice (rosa26-CAG-loxp-stop-loxp-FCN3 knock-in, FCN3^flox/flox^) were generated by Nanjing Biomedical Research Institute of Nanjing University (Jiangsu) using the CRISPR–Cas9 system. The mouse specifically overexpressing FCN3 in the liver (FCN3^LKI^) were generated by crossing FCN3^flox/flox^ with albumin-Cre mice (The Jackson Laboratory). To establish an HCC model, eight-week-old FCN3^LKI^ mice and their littermate controls were subjected to a high-fat diet (HFD, with 60 kcal% fat, D12492, New Brunswick) and a high sugar solution (23.1 g/L d-fructose (G8270, Sigma-Aldrich) and 18.9 g/L d-glucose (F0127, Sigma-Aldrich)) for 16 weeks. Subsequently, mice were intraperitoneally injected with CCl_4_ (289116, Sigma-Aldrich) at a dose of 0.5 ml/kg of body weight (dissolved in corn oil) twice weekly, concurrently with the same dietary regimen. The onset of HCC was observed at both 28th and 32th weeks following the combined CCl_4_ and dietary induction [24]. The mice were euthanized by narcotic drugs overdose in accordance with the recommendations for the euthanasia of experimental animals. During our study, we observed an 8% mortality rate; the deceased mice were omitted from the tumor incidence analysis. The mice were kept under specific pathogen-free (SPF) conditions with a 12 h light/dark cycle. The protocol was approved by the Animal Care and Use Committees of Shanghai Sixth People’s Hospital Affiliated to Shanghai Jiao Tong University School of Medicine.

### Cell culture

The human HCC cell lines (Huh7, HepG2) were obtained from the cell bank of the Type Culture Collection of the Chinese Academy of Sciences. HEK-293T and HCC cell lines (SM-386, YY-8103, MHCC97-H) were obtained from ATCC cell bank, and routinely maintained in Dulbecco’s modified Eagle’s medium (DMEM, Gibco) supplemented with 10% fetal bovine serum (FBS, Gibco). L02 cells were purchased from Shanghai Zhong Qiao Xin Zhou Biotechnology Co. Ltd., and cultured in Roswell Park Memorial Institute (RPMI) 1640 medium (Gibco) supplemented with 10% FBS. All cells were maintained in a humidified 5% CO_2_ atmosphere at 37 °C, and tested for mycoplasma contamination every month. FCN3-overexpressed MHCC97-H, YY-8103 and control cells were treated with T0901317 (HY-10626, MedChemExpress), Erastin (HY-15736, MedChemExpress), FINO2 (HY-129457, MedChemExpress), AKTi-1/2 (HY-10355, MedChemExpress), Ferrostatin-1 (SML0583, Sigma-Aldrich), N-acetyl-L-cysteine (A9165, Sigma-Aldrich), Z-VAD-fmk (FMK001, R&D Systems), and Necrostatin-1 (HY-15760, MedChemExpress) for indicated time.

### Human samples

The pan-cancer tissue microarray (TMA) and all tumor, PVTT and paired adjacent normal tissues were collected from HCC patients. All patients signed informed consent forms, and their clinical information was collected from medical records, which was list in Table S1. All experiments were approved by Zhongshan Hospital (project approval number B2023-291).

### Plasmid construction

The isoforms of FCN3 complementary DNA (cDNA, NM_173452.3) were obtained by PCR from hepatic cDNA and cloned into a lentivirus vector (pLVX-IRES-puro). The no-secretory mutant of FCN3 (nsFCN3, Δaa 2–23) was subcloned from the FCN3 with the deletion of N-terminal signal peptide sequence. For protein purification, FCN3 was subcloned into pcDNA3.1 vector with a His tag. The pro-IR cDNA was subcloned into the pcDNA3.1 with a C-terminal HA-tag. Furin-Flag plasmid was purchased from Miao Ling Biotechnology Co. Ltd (P36961). We have provided maps of the plasmids in Table S2.

The FCN3-specific shRNA was designed by the BLOCK-iT™ RNAi Designer (ThermoFisher Scientific). To construct the pLKO.1-FCN3-shRNA plasmids, the forward and reverse oligos were annealed and cloned to the pLKO.1 plasmid vector. The oligonucleotide sequences were listed in Table S3.

### Lentivirus preparation and stable expression cell line construction

To acquire the lentivirus, the pLVX-FCN3 or pLKO.1-shFCN3 plasmids were co-transfected with lentiviral package vectors (psPAX and pMD2.G) into HEK-293T cells for lentivirus generation using Lipofectamine™ 3000 Reagent (ThermoFisher Scientific) following manufacturer’s instructions. At 48 h posttransfection, virus-containing supernatant of HEK-293T cells was harvested. The lentivirus was enriched by centrifugation at 20,000 rpm for 2 h. After removing the supernatant, the precipitate containing lentivirus particles was resuspended with DMEM.

To construct the stable expression cell lines, the packaged lentivirus was added into the culture medium of the indicated HCC cells and maintained for 8 h. The stable expression cells were selected by treating with puromycin for 3 days or longer.

### Real-time PCR

Cellular RNA was isolated from different HCC cells with TRIzol reagent (15596, Life Technologies) following manufacturer’s instructions. Then, reverse transcription was performed from RNA to cDNA with ABScript III RT Master Mix (RK20428, Abclonal). Finally, PCR was performed with 2 × Universal SYBR Green Fast qPCR Mix (RK21203, Abclonal) on a specific PCR instrument (Roche Life Science). The real-time PCR primers were designed to span an intro-exon junction in order to avoid genomic DNA amplification, and their details were listed in Table S3.

### Triglyceride (TG) assay

TG levels in HCC cells treated with PA (100 µM) for 24 h were detected with a commercial kit (E1013–105, Applygene). Briefly, six-well plate-cultured cells were collected in 200 µL of lysis buffer. The lysate was kept still for 10 min. The supernatant was placed into a new centrifuge tube and heated for 10 min at 70 °C. After centrifugation at 2,000 rpm for 5 min, the supernatant was taken to detect the TG level at a wavelength of 550 nm in a SpectraMax i3 Multi-Mode Detection instrument (Molecular Devices). Finally, the value was normalized to the protein concentration, which was quantified with a BCA reagent (P0012, Beyotime).

### Cellular metabonomic analysis by LC‒MS

The metabolites were analyzed by LC‒MS assay. Briefly, the indicated HCC cells were seeded in 6-cm dishes and grown to 80–90% confluence. After washing with ice-cold normal saline twice, the cells were scraped off with cell scraping. The precipitates were collected after centrifugation at 2,000 rcf for 5 min and frozen with liquid nitrogen. The cells were dissolved in 300 µL pretreatment reagent (methanol : acetonitrile : water = 2 : 2 : 1, containing chlorophenylalanine 2 µg/mL) and ground for 2 min at 4 °C. After centrifugation at 12,000 rpm for 20 min at 4 °C, 260 µL of supernatant was concentrated to dry by centrifugation at room temperature and under vacuum for 1–2 h at 45 °C. The dry powder was dissolved in 50–100 µL of resoluble reagent (methanol : water = 3 : 7). After centrifugation at 12,000 rpm for 20 min at 4 °C, 40 µL of supernatant was placed into a vial for measurement. Global metabolism profiling was performed on the ACQUITY UPLC HSS T3 (100 × 2.1 mm, 1.7 μm, waters). MS parameters were set as follows: the mode was the HESI source in positive and negative mode capillary; the full scan range was 67 to 1000 amu; the full scan resolution was 70000, AGC (1e6), IT (100 ms); the spray voltage was 3.2 kV (positive mode) and 2.8 kV (negative mode); and the capillary temperature was 320 °C. The data were acquired by Xcalibur 3.0 software (ThermoFisher Scientific) and processed by Progenesis QI v2.3 data analysis software (Waters). Further statistical analysis was performed with EZinfor V3.0.3 (Umetrics). Metabolites meeting the following conditions were defined as differential metabolites: ANOVA (p) < 0.05, multiple changes (fold change) > 1.5, OPLSDA model VIP > 1, coefficient of metabolite variation CV% < 30%, and identified after searching the database.

### RNA sequencing

Cellular RNA was isolated from nsFCN3-overexpressed and control MHCC97-H cells (*n* = 3). The RNA was quantified with a Qubit 4.0 instrument (Invitrogen), and the quality was examined with agarose gel electrophoresis, with the brightness of 28S/23S higher than that of 18S/16S. RNA sequencing libraries were constructed with 500 ng of RNA using the VVAHTS® Universal V8 RNA-seq Library Prep Kit for Illumina (NR605-0, Vazyme) following the manufacturer’s instructions. The sequencing process was accomplished on an Illumina NovaSeq 6000 system.

### Western blot

Total proteins were collected from the lysates of HCC tissues or cells with RIPA buffer combined with protease and phosphatase inhibitor cocktail (k1008 & k1015, APExBIO). Protein concentrations were measured with BCA reagent. The protein was separated by SDS‒PAGE and then transferred onto a PVDF membrane (ISEQ00010, Millipore). After blocking with 5% BSA for 1 h at room temperature, the membranes were incubated with primary antibody overnight at 4 °C, followed by incubation with peroxidase-conjugated secondary antibody for 1 h at room temperature. The antibodies were listed with the dilution, catalogue number, and supplier: FCN3, 1:1000, AF2367, R&D Systems; GPX4, 1:1000, 67763-1-lg, Proteintech; SREBP1c, 1:1000, 14088-1-AP, Proteintech; FASN, 1:1000, 10624-2-AP, Proteintech; SCD1, 1:1000, ab236868, Abcam; E-Cadherin, 1:1000, #3195, CST; N-Cadherin, 1:1000, #13116, CST; Slug, 1:1000, #9585, CST; Vimentin, 1:1000, 10366-1-AP, Proteintech; ACC, 1:1000, #3676, CST; p-AKT (S473), 1:1000, #4060, CST; AKT, 1:1000, #4691, CST; IR-β, 1:1000, #23413, CST; p-IGF-IR, 1:1000, #3024, CST; ACSL4, 1:1000, 22401-1-AP, Proteintech; NRF2, 1:1000, A0674, ABclonal; PTP1B, 1:1000, 11334-1-AP, Proteintech; Furin, 1:1000, 18413-1-AP, Proteintech; phosphatase and tensin homolog (PTEN), 1:1000, #9188, CST; protein phosphatase 2 (PP2A) A subunit, 1:1000, #2041, CST; PP2A C subunit, 1:1000, #2259, CST; HA, 1:2000, M2003, Abmart; β-Tubulin, 1:3000, AP0064, Bioworld; GAPDH, 1:3000, A19056, ABclonal; β-Actin, 1:3000, #3700, CST; Hsp90, 1:1000, #4874, CST; Apoptosis antibody sampler kit, #9915, CST; HRP Goat Anti-Rabbit IgG (H + L), 1:50000, E030120, Earthox; HRP Goat Anti-Mouse IgG (H + L), 1:50000, E030110, Earthox; HRP Donkey Anti-Goat IgG (H + L), 1:5000, AS031, ABclonal.

### Histological staining

Tumors or livers were paraformaldehyde-fixed, paraffin-embedded and cut into 5 μm thick slices. For immunohistochemical (IHC) staining, the deparaffinated sections were subjected to antigen retrieval and boiled for 15 min in 10 mM/L citrate buffer (pH = 6.0). Then, the sections were blocked with 5% BSA for 30 min, incubated with primary antibody Ki67 (ab16667, 1:200 dilution, Abcam), ACSL4 (22401-1-AP, 1:200 dilution, Proteintech), FTH1 (A19544, 1:200 dilution, ABclonal), PTGS2 (ab179800, 1:100 dilution, Abcam), FCN3 (11867-1-AP, 1:100 dilution, Proteintech) overnight, incubated with a biotinylated anti-rabbit IgG antibody (1 : 400) for 1 h, and treated with a DAB peroxidase substrate kit (SK-4100, Vector Laboratories Inc.). Finally, the sections were processed with hematoxylin staining. TMA chip was further photographed and scored by Vectra 2 (Perkinelmer). All quantifications were evaluated blinded to patient clinical outcomes. For Sirius red staining, the sections were immersed in a staining agent for collagen coloration. Subsequently, they were dehydrated and made transparent using a gradient of alcohol and xylene. Finally, the sections were sealed with neutral gum and prepared for microscope photography.

### Immunoprecipitation (IP)

The cells were lysed with IP lysis buffer. After centrifuging, the supernatant was taken into a new tube, and incubated with indicated antibodies at 4 ˚C overnight. Then, 30 µL protein A/G sepharose beads (P2055, Beyotime) was washed with IP lysis for 3 times, and incubated with the mixture above for plus 3 h. Subsequently, the beads were pelleted down and adequately washed 3 times with IP lysis buffer. Finally, the beads were boiled in 2 × loading buffer for western blotting analysis.

### Protein purification

FCN3-His vectors were transfected into HEK-293E cells. Six days later, cell culture medium was collected and incubated with Ni-NTA (Qiagen) beads at 4 °C overnight. The incubated beads were pelleted down, and adequately washed, followed by eluting with elution buffer (50 mM Tris, 500 mM NaCl, 250 mM Imidazole, pH 8.0). The protein concentration in the elution buffer was measured with BCA reagent.

### In vitro cell growth assay

Crystal violet and CCK8 assays were used to examine cell growth in vitro.

For crystal violet staining, 1 × 10^3^ cells of each cell line were seeded in 6-well plates. When the cell clone grew to the point of being visible to the naked eye, 0.1% crystal violet solution was used to stain the cell clone for 5 min. The plate was allowed to dry, and photographs were taken.

For the CCK8 assay, 1 × 10^3^ cells of each cell line were seeded in 96-well plates (three multiple wells). Every 24 h, the absorbance was measured at a wavelength of 450 nm in a SpectraMax i3 Multi-Mode Detection instrument (Molecular Devices), after the CCK8 solution (40203ES88, Yeasen) adding into the medium for 2 h.

For cell viability assays, 3 × 10^3^ cells per well were seeded in 96-well plates (three multiple wells). Cells were treated with the indicated concentrations of erastin and FINO2 for 24 h. Then the CCK8 value was measured, and the cell viability of the samples was calculated according to the manufacturer’s instructions.

For the other cell viability assay, 2 × 10^5^ different HCC cells were seeded in 6-well plates. The cells with approximately 50% confluence were treated with the indicated concentrations of erastin and FINO2 for 24–48 h. Then, the cells were photographed with a light microscope (Olympus). After being photographed, the cells were digested, collected, and resuspended in 100 µL DMEM. Subsequently, 100 µL of 0.4% trypan blue solution were mixed with the cell suspension and incubated for 5 min. The mixture was counted with a hemocytometer and photographed with a light microscope (Olympus). The dead cells were stained blue, while the viable cells remained clear.

### Lipid peroxidation analysis

For detection of ROS, nsFCN3-overexpressed and control MHCC97-H/YY-8103 cells were plated into 6-cm chamber slide. The cells were treated with erastin for 12 h, and incubated with 10 µM BODIPY-581/591 C11 (D3861, Invitrogen) for 30 min. Then the cells were washed with PBS and photographed with a confocal laser scanning microscope (ZEISS). Upon oxidation in live cells, fluorescence shifts from red to green of the phenylbutadiene segment of the fluorophore, providing a ratiometric indication of lipid peroxidation.

### Malondialdehyde (MDA) assay

The extent of lipid peroxidation in HCC cells was detected with the MDA assay kit (S0131, Beyotime) according to the manufacturer’s instructions. In brief, the thiobarbituric acid (TBA) was configured as a 0.37% solution. The MDA-TBA solution was prepared with MDA and TBA diluent (1 : 3) added with antioxidants. Six-well plate-cultured cells were lysed with 100 µL of RIPA lysis buffer. After centrifuging at 10,000 rpm for 10 min, the supernatant was transferred into a new centrifuge tube. Then 200 µL of MDA-TBA solution and 100 µL supernatant were mixed, and heated at 100 °C for 15 min. After centrifugation at 1,000 rcf for 10 min, 200 µL of the mixture was taken to detect the MDA level at a wavelength of 532 nm in a SpectraMax i3 Multi-Mode Detection instrument (Molecular Devices). Finally, the MDA value was normalized to the protein concentration, which was quantified by a BCA reagent.

### Flow cytometry analysis

The ferroptosis of HCC cell were analyzed with the Annexin V-PE/7-AAD apoptosis detection kit (40310ES60, Yeasen) [25]. In brief, the cells underwent digestion with serum-free EDTA and were then washed twice with pre-cooled PBS at 4 °C, followed by centrifugation for 5 min on each occasion. Next, the cells were suspended in 250 µL of flow cytometry binding buffer. Subsequently, 100 µL of the cell suspension was transferred to a 5 mL test tube, to which 10 µL of 7-AAD and 5 µL of Annexin V/PE staining solution were added. The cells were gently vortexed and left to incubate in the dark at room temperature (25 °C) for 15 min. Following this, 400 µL of binding buffer was added to each tube. Finally, the cell suspension was processed using the BD FACS Melody instrument, and the resulting data were analyzed.

### Trans-well assay

For the trans-well assay, a certain amount of HCC cells was resuspended in 100 µL DMEM and placed into the upper chamber of a noncoated membrane (Corning, pore size: 8 μm), while the lower chamber was filled with 500 µL DMEM containing 10% FBS. After the cells migrating through the membrane pores for indicated time, the upper cells in the membrane surface were wiped off with a cotton swab. The migrated cells at the lower surface of the membrane were fixed with 4% paraformaldehyde for 25 min, followed by staining with 0.1% crystal violet solution for 15 min. The cells were washed with clean water to remove the floating color. The membrane was then photographed with a light microscope (Olympus). The number of migrated cells was counted with Adobe Photoshop CS5.

### Statistical analysis

Correlation analyses in human HCC/PVTT tissues and TCGA database utilized the Spearman correlation coefficient. Differences between unpaired samples were assessed using the unpaired Student’s *t* test (for normally distributed data) or the Mann-Whitney *U* test (for data not normally distributed). Mean values across multiple groups were evaluated using one-way analysis of variance (ANOVA). Comparisons between paired samples from HCC or PVTT and adjacent non-tumor tissues were conducted using the paired Student’s *t* test (for normally distributed data) or the Wilcoxon matched-pairs signed rank test (for data not normally distributed). Log-rank test was applied to analyze survival curve. Data are presented as the mean ± Standard Deviation (SD). For TMA correlation analysis, Pearson’s χ2 test was used to acquire *p* value, and *s* = − log_2_(*p*) [26]. Mice are grouped randomly and histological analysis were performed blindly. The sample size in each experiment was determined based on previous studies and preliminary results. Outliers were excluded prior to statistical analysis.

## Results

### FCN3 enhances the sensitivity of HCC cells to ferroptosis

**Fig. 1:**
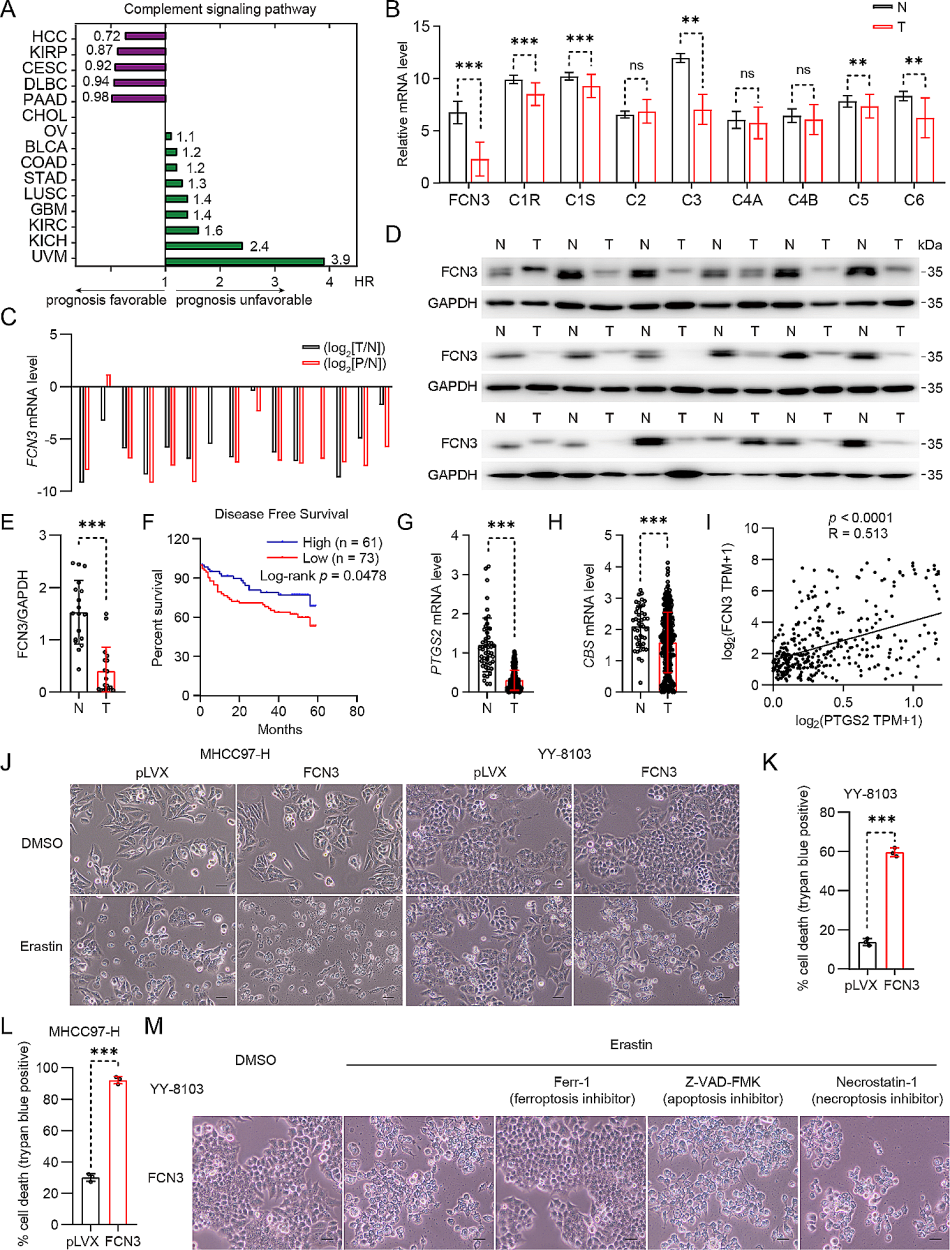
The reduced FCN3 level in HCC are associated with ferroptosis resistance (**A**) The complement signaling pathway associated with prognosis (green represents unfavorable, purple represents favorable) in various tumors was analyzed by Kaplan–Meier analysis based on TCGA database. (KIRP: Kidney renal papillary cell carcinoma, CESC: Cervical squamous cell carcinoma, DLBC: Lymphoid Neoplasm Diffuse Large B-cell Lymphoma, PAAD: Pancreatic adenocarcinoma, CHOL: Cholangio carcinoma, OV: Ovarian serous cystadenocarcinoma, BLCA: Bladder Urothelial Carcinoma, COAD: Colon adenocarcinoma, STAD: Stomach adenocarcinoma, LUSC: Lung squamous cell carcinoma, GBM: Glioblastoma multiforme, KIRC: Kidney renal clear cell carcinoma, KICH: Kidney Chromophobe, UVM: Uveal Melanoma). (**B**) mRNA expression of complement-related genes in normal human liver tissues (**N**) and HCC tissues (T) in published data from TCGA database. (**C**) mRNA expression of *FCN3* in 15 groups of HCC (T), PVTT (P) and paired adjacent nontumor (**N**) tissues. log_2_(T/N) value < 0 indicated that *FCN3* expression was downregulated, while log_2_(T/N) value > 0 indicated that *FCN3* expression was upregulated in HCC and PVTT samples. (**D**-**E**) Immunoblot (**D**) and quantification (**E**) of FCN3 in 18 pairs of HCC (T) and adjacent nontumor (N) tissues. GAPDH was used as a loading control. FCN3 exhibited two bands in human samples (two transcripts). (**F**) Kalpan–Meier analysis of disease-free survival of tissue microarray (TMA) data containing 134 patients. (**G**-**H**) mRNA expression of *PTGS2* (**G**) and *CBS* (**H**) in normal human liver tissues (N) and HCC tissues (**T**) in data from TCGA database. (**I**) Correlation analysis of *PTGS2* and *FCN3* expressions based on TCGA database. (**J**) Representative images of FCN3-overexpressed YY-8103 and MHCC97-H cells treated with erastin. Scale bar, 100 μm. (**K**-**L**) Quantification of trypan blue staining for death cell in FCN3-overexpressed YY-8103 (**K**) and MHCC97-H (**L**) cells treated with erastin in (**J**). (**M**) FCN3-overexpressed YY-8103 cells were treated with erastin (10 µM) and specific cell death inhibitors. Scale bar, 100 μm Data are from one representative experiment of three independent experiments (C-D and J-M). Data are presented as mean ± SD. Significance was assessed by Mann-Whitney *U* test (B, E, G, H), Log-rank test (**F**), Spearman correlation (**I**), Student’s *t* test (K, L). **p < 0.01, ***p < 0.001 compared with the control group

To comprehensively explore the involvement of the complement system in HCC development, we performed a systematic bioinformatics analysis of the complement system members using Pathcards data (https://pathcards.genecards.org) and TCGA pancancer transcriptomics data (http://gepia2.cancerpku.cn). Our investigation has unveiled a noteworthy association between the complement signaling pathway and a favorable prognosis in specific cancer types, among which HCC displayed the most pronounced link to this pathway (Fig. 1A). Moreover, we detected a substantial downregulation of key components within the complement system in HCC, with FCN3 emerging as the most under-expressed member (*p*(HR) < 0.05) (Fig. 1B; Table S4). We further assessed FCN3 expression across 15 discrete groups, comprising HCC tumor specimens (T), their corresponding adjacent non-tumor tissues (N), and portal vein tumor thrombus (P, PVTT). Our analysis unveiled a significant reduction in FCN3 expression within HCC and PVTT, compared to the control groups (Fig. 1C), which was consistent with the previous report [23]. In line with this, immunoblotting and immunohistochemistry analysis also confirmed the reduction of FCN3 in human HCC (Fig. 1D, E; Fig. S1A), which was consistent with reported results [27].

To explore the clinical significance of FCN3 in HCC, we examined FCN3 expression in 134 tumor specimens from an HCC tissue microarray with FCN3 antibody staining (Fig. S1B). Our analysis revealed that patients with lower FCN3 levels showed poorer disease-free survival (*p* = 0.0478) compared to those with higher FCN3 levels (Fig. 1F). Further examination indicated that lower FCN3 expression was significantly correlated with higher alpha-fetoprotein (AFP) level, larger tumor size, higher TNM stage, and earlier recurrence (Table 1). Moreover, analysis of the TCGA dataset demonstrated a strong correlation between low FCN3 expression in HCC patients and poorer overall survival (*p* = 0.032) (Fig. S1C), as well as inferior disease-free survival (*p* = 0.0083) (Fig. S1D). Collectively, these findings suggest that reduced FCN3 expression may be associated with the progression of HCC.

**Table 1:** The correlation between the fcn3 and clinicopathologic parameters in HCC patients

Characteristic	High Expression	Low Expression	*p* value	s value
	Group (*n* = 61)	Group (*n* = 73)		
**Gender**				
Male	52	60	0.635	0.66
Female	9	13		
**Age (years)**				
< 65	45	57	0.560	0.84
≥ 65	16	16		
**Liver Cirrhosis**				
No	33	44	0.472	1.08
Yes	28	29		
**AFP (ng/mL)**				
≤ 400	55	55	0.023*	5.44
> 400	6	18		
**Tumor Size (cm)**				
≤ 5	54	42	< 0.0001***	13.29
> 5	7	31		
**Tumor Number**				
Single	53	61	0.591	0.76
Multiple	8	12		
**Satellite Nodules**				
No	60	69	0.243	2.04
Yes	1	4		
**MVI stage**				
2	4	11	0.120	3.06
0/1	57	62		
**TNM stage**				
I/II	38	32	0.033*	4.92
III/IV	23	41		
**Recurrence**				
No	47	44	0.038*	4.72
Yes	14	29		
**Survival status**				
Survival	53	61	0.591	0.76
Death	8	12		

HCC hepatocellular carcinoma

*p < 0.05, **p < 0.01, ***p < 0.001 values are set for highly significant differences

*s* value *s* = − log_2_(*p*)

Conversely, we detected an obvious reduction in the expression of *PTGS2* and *CBS*, two ferroptosis markers [28], in HCC and PVTT (Fig. 1G, H; Fig. S1E). Notably, we observed that there is a significant positive correlation between the levels of FCN3 and PTGS2, as well as between FCN3 and CBS in patients with HCC (Fig. 1I; Fig. S1F). These observations led us to hypothesize that FCN3 plays a pivotal role in the process of ferroptosis within HCC. To investigate this hypothesis, we first established stable cell lines overexpressing FCN3 through lentivirus infection (Fig. S1G) and subsequently subjected them to erastin, a widely used inducer of ferroptosis. Our findings revealed that the HCC cells overexpressing FCN3 were more susceptible to cell death after treated with erastin, compared to their corresponding control (Fig. 1J-L). To confirm the mode of erastin-induced cell death, we treated cells with ferrostatin-1 (Ferr-1), a specific inhibitor of ferroptosis. Notably, Ferr-1 substantially blocked cell death in the HCC cells overexpressing FCN3 (Fig. 1M). In contrast, inhibitors of other forms of cell death, including apoptosis (Z-VAD-FMK) and necroptosis (Necrostatin-1), failed to suppress erastin-induced cell death (Fig. 1M).

Taken together, our findings indicate that FCN3 increases the sensitivity of HCC cells to ferroptosis.

### FCN3 induces ferroptosis in HCC cells through its intracellular functionality

Next, we aimed to explore the impact of FCN3-promoted ferroptosis on the migration and survival of HCC cells. Firstly, we assessed FCN3 expression in diverse HCC cell lines (Fig. S1H, I). Consistently, FCN3 expression was downregulated in numerous HCC cell lines, including SM-386, YY-8103, MHCC97-H, HepG2 and Huh7, compared to the normal human hepatocyte cell line L02 (Fig. S1I).

**Fig. 2:**
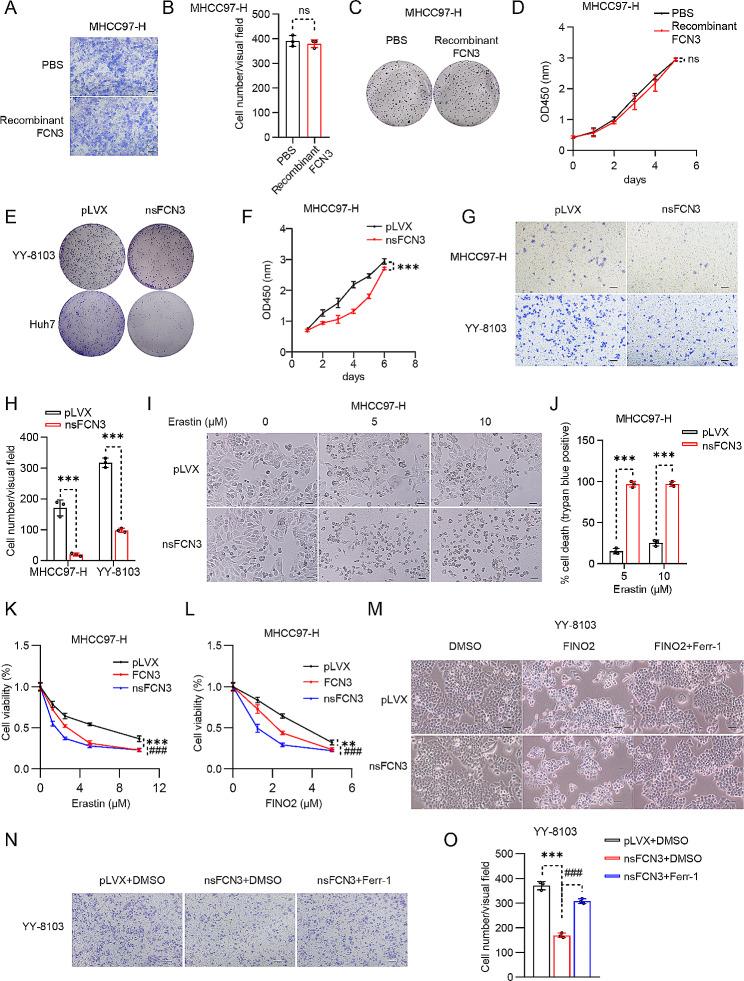
Intracellular FCN3 inhibits the migration and survival of HCC cells by promoting ferroptosis (**A**-**B**) Representative images (**A**) and quantification (**B**) of migrating MHCC97-H cells after treatment with 300 ng/mL FCN3 recombinant protein. Scale bar, 100 μm. (**C**-**D**) Crystal violet (**C**) and CCK8 assay (**D**) in FCN3 recombinant protein treated MHCC97-H cells, the treatment medium was refreshed every three days, FCN3 recombinant protein: 300 ng/mL. (**E**) Representative images of the crystal violet assay in nsFCN3-overexpressed YY-8103 and Huh7 cells. (**F**) CCK8 assay in nsFCN3-overexpressed MHCC97-H cells. (**G**) Representative images of migrating MHCC97-H and YY-8103 cells that cultured in trans-well plates. Scale bar, 100 μm. (**H**) Quantification of the average number of migrating MHCC97-H and YY-8103 cells in (**G**). (**I**) Representative images of nsFCN3-overexpressed MHCC97-H cells treated with erastin. Scale bar, 100 μm. (**J**) Quantification of trypan blue staining for death cell in nsFCN3-overexpressed MHCC97-H cells treated with erastin. (**K**-**L**) Viability of FCN3- and nsFCN3-overexpressed MHCC97-H cells treated with indicated concentrations of erastin (**K**) or FINO2 (**L**) for 24 h. (**M**) Representative images of nsFCN3-overexpressed YY-8103 cells treated with FINO2 (10 µM) and Ferr-1 (2 µM). Scale bar, 100 μm. (**N**-**O**) Representative images (**N**) and quantification (**O**) of migrating YY-8103 cells treated with Ferr-1 (2 µM). Scale bar, 100 μm Data are presented as mean ± SD and are from one representative experiment of three independent experiments. Significance was assessed by Student’s *t* test (B, H, J, O). **p < 0.01, ***^, ###^*p* < 0.001 compared with the control group. ns, not significant

Given that FCN3 is a secreted protein, we initially purified recombinant FCN3 protein and treated MHCC97-H cells with this recombinant protein. Astonishingly, recombinant FCN3 had no detectable impact on the growth and migration of HCC cells (Fig. 2A-D). However, the stable cell lines overexpressing FCN3 exhibited a noteworthy inhibition in migration (Fig. S1J, K) and growth (Fig. S1L) in the HCC cells overexpressing FCN3. Conversely, FCN3 knockdown promoted colony formation (Fig. S1M), and enhanced cell growth (Fig. S1N) in Huh7 cells.

The disparity between the outcomes from recombinant protein treatment and the stable transgenic cell lines suggests that FCN3 may exert its anti-cancer effects via its intracellular function. To further explore this, we generated a lentivirus expressing a mutant FCN3 with its secretory signal peptide deleted (nsFCN3) and subsequently overexpressed nsFCN3 in HCC cells with this viral construct (Fig. S1G). Notably, we found that both nsFCN3 and FCN3 inhibited HCC growth and migration, with nsFCN3 exhibiting a more potent effect (Fig. S1J-L). To re-confirm these observations, we conducted a set of analogous experiments with other different HCC cells lines overexpressing nsFCN3. Consistently, these trials revealed a significant inhibition in both cell growth (Fig. 2E, F; Fig. S1O, P) and migration (Fig. 2G, H) in the HCC cells overexpressing nsFCN3. Collectively, these findings underscore the pivotal role of intracellular FCN3 in suppressing the growth and migration of HCC cells.

Subsequently, HCC cells with nsFCN3 overexpression were subjected to erastin treatment, consistently revealing that nsFCN3 also enhances the susceptibility of HCC cells to ferroptosis (Fig. 2I; Fig. S2A). This was re-confirmed by trypan blue staining, showing increased cell death in the HCC cells overexpressing nsFCN3 in the presence of erastin, compared to the control group (Fig. 2J; Fig. S2B-D). Consistently, overexpressing FCN3 also led to a greater extent of cell death in HCC cells when treated with another ferroptosis inducer, FINO2 (Fig. S2E-G). However, recombinant FCN3 protein treatment had no obvious effect on ferroptosis susceptibility (Fig. S2H, I). Furthermore, in the presence of varying doses of erastin or FINO2, both FCN3- or nsFCN3-expressing HCC cells exhibited reduced viability, with nsFCN3 overexpression leading to a higher level of cell death (Fig. 2K, L; Fig. S2J). Flow cytometry analysis also revealed that HCC cells overexpressing nsFCN3 were more prone to undergo ferroptosis, as indicated by 7-AAD staining, compared to the control cells (Fig. S2K, L). Conversely, the nsFCN3-caused ferroptosis in presence of FINO2 was substantially blunted by Ferr-1, a ferroptosis inhibitor (Fig. 2M). Moreover, Ferr-1 effectively counteracted the suppressive effects induced by nsFCN3 in the migration of HCC cells (Fig. 2N, O).

In conclusion, these findings support the notion that FCN3 promote ferroptosis in HCC cells, owing to its intracellular function.

### FCN3 overexpression suppresses HCC development in vivo

**Fig. 3:**
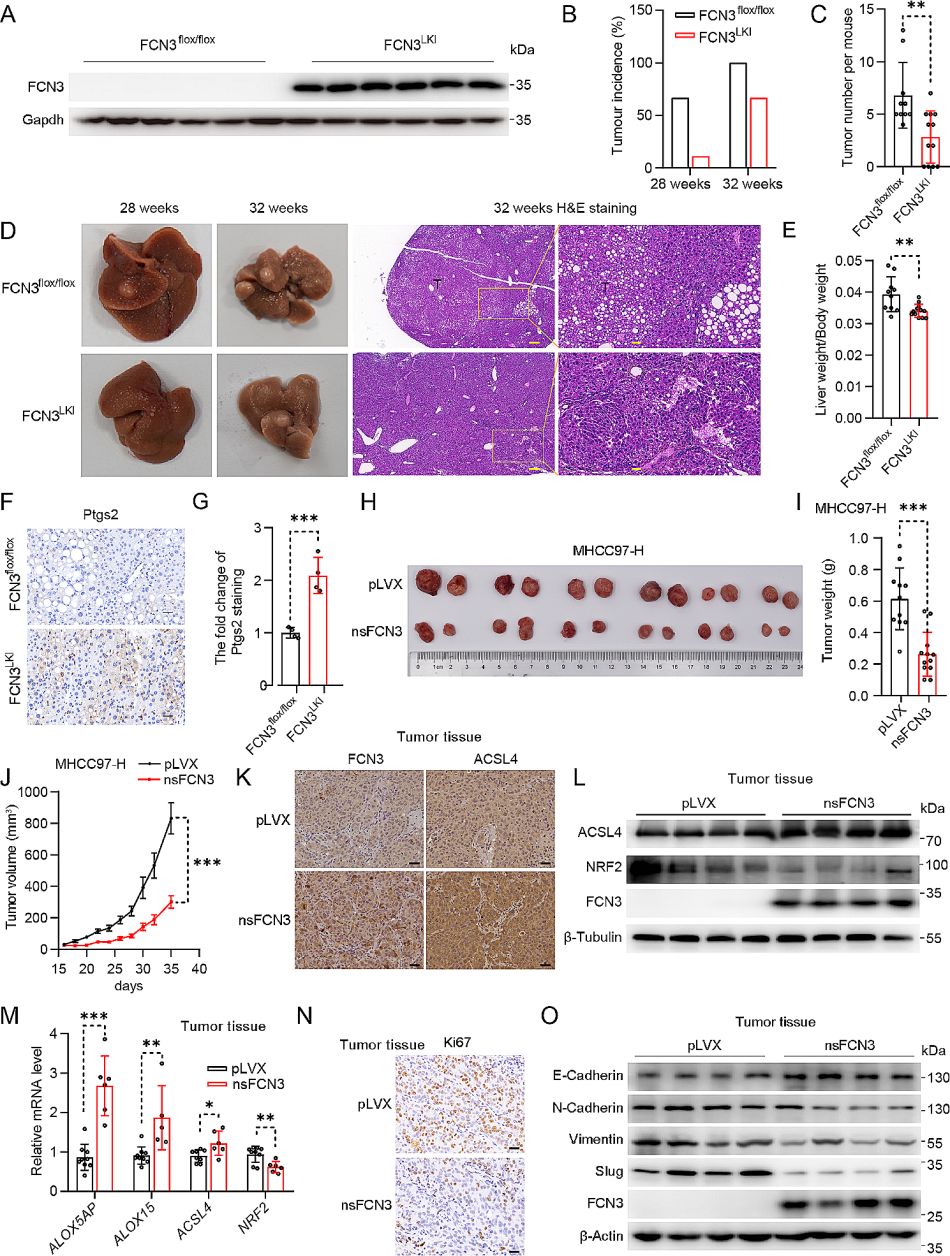
FCN3 inhibits the development of HCC in vivo (**A**) Immunoblot of FCN3 in the liver of FCN3^LKI^ and control mice. Gapdh was used as a loading control. (**B**) Tumor incidence from FCN3^LKI^ and control mice inducted for 28 and 32 weeks. (**C**) Quantification of total surface tumors number from FCN3^LKI^ and control mice inducted for 32 weeks. (**D**) Representative images of the livers and tumors from FCN3^LKI^ and control mice inducted for 28 and 32 weeks. Scale bar, 200 μm & 40 μm. (**E**) Ratios of liver weight to body weight at week 32. (**F**) Representative images of Ptgs2 staining in mouse liver. Scale bar, 20 μm. (**G**) Quantification of Ptgs2 staining in (**F**). (**H**) Representative image of xenografts for nsFCN3-overexpressed and control MHCC97-H cells at day 36 (*n* = 6 mice per group). (**I**) Weights of xenograft tumors in (**H**) at day 36 (*n* = 12 per group). (**J**) A subcutaneous tumor volume curve from (**H**) (*n* = 6 mice per group). (**K**) Representative images of FCN3 and ACSL4 IHC staining in xenograft tumors. Scale bar, 40 μm. (**L**) Immunoblots of ACSL4 and NRF2 in xenograft tumors. β-Tubulin was used as a loading control. (**M**) mRNA levels of ferroptosis-related genes in xenograft tumors. (**N**) Representative images of Ki67 IHC staining in xenograft tumors. Scale bar, 20 μm. (**O**) Immunoblots of EMT-related proteins in xenograft tumors. β-Actin was used as a loading control Data are presented as mean ± SD. Significance was assessed by Mann-Whitney *U* test (C), Student’s *t* test (E, G, I, M). *p < 0.05, **p < 0.01, ***p < 0.001 compared with the control group

Next, we aim to investigate the in vivo anticancer effects of FCN3 using mouse models with primary hepatocellular carcinoma or subcutaneous HCC xenografts. Firstly, we generated a knock-in mouse model specifically overexpressing human FCN3 in the liver (FCN3^LKI^) (Fig. 3A; Fig. S3A). The FCN3^LKI^ mice and their littermate controls (FCN3^flox/flox^) were then subjected to a HFD diet, high sugar drinks water, and CCl_4_-treatment to induce HCC (Fig. S3B) [24]. Notably, the liver-specific overexpression of FCN3 reduced the incidence of hepatocellular carcinoma (Fig. 3B; Fig. S3C). Moreover, FCN3^LKI^ mice exhibited a significant reduction in tumor burden (Fig. 3C, D) along with a notable decrease in the liver weight-to-body weight ratio, as compared to the control mice (Fig. 3E). There was no significant difference in body weight between the two groups (Fig. S3D). In addition, FCN3^LKI^ mice also exhibited downregulated mRNA levels of genes associated with inflammatory responses and fibrosis in comparison to the control mice (Fig. S3E, F). Consistently, FCN3^LKI^ mice showed a lower degree of fibrosis, as indicated by Sirius red staining (Fig. S3G, H). Notably, Ptgs2 expression was significantly upregulated in the FCN3 overexpression group (Fig. 3F, G).

Next, we established an HCC xenografts mouse model by subcutaneously injecting MHCC97-H cells overexpressing nsFCN3 and mock cells into athymic mice. Consistently, nsFCN3 overexpression significantly retarded tumor growth (Fig. 3H-J). We further explored the effect of FCN3 on ferroptosis in the xenograft tumors. IHC staining revealed a substantial increase in the expression of ACSL4, a ferroptosis biomarker [28], in nsFCN3-overexpressed xenograft tissue compared to control (Fig. 3K). Consistently, immunoblotting analysis confirmed the elevated ACSL4 expression (Fig. 3L), as well as a significant decrease in the expression of NRF2 (Fig. 3L), whose level is negatively associated with ferroptosis [29]. Furthermore, qPCR analysis for the tumor tissue revealed that overexpression of nsFCN3 significantly increased the expression of lipid peroxidation enzymes and downregulated the level of *NRF2* (Fig. 3M). Additionally, the tumors overexpressing nsFCN3 exhibited reduced proliferation as evidenced by Ki67 immunohistochemical staining (Fig. 3N; Fig. S3I), as well as suppressed EMT process (Fig. 3O; Fig. S3J). Besides, apoptosis was slightly heightened in tumors overexpressing nsFCN3 compared to the control group (Fig. S3K).

In summary, these findings underscore the critical role of FCN3 in promoting ferroptosis and suppressing the progression of HCC in vivo.

### FCN3 sensitizes HCC cells to ferroptosis by decreasing MUFA levels

**Fig. 4:**
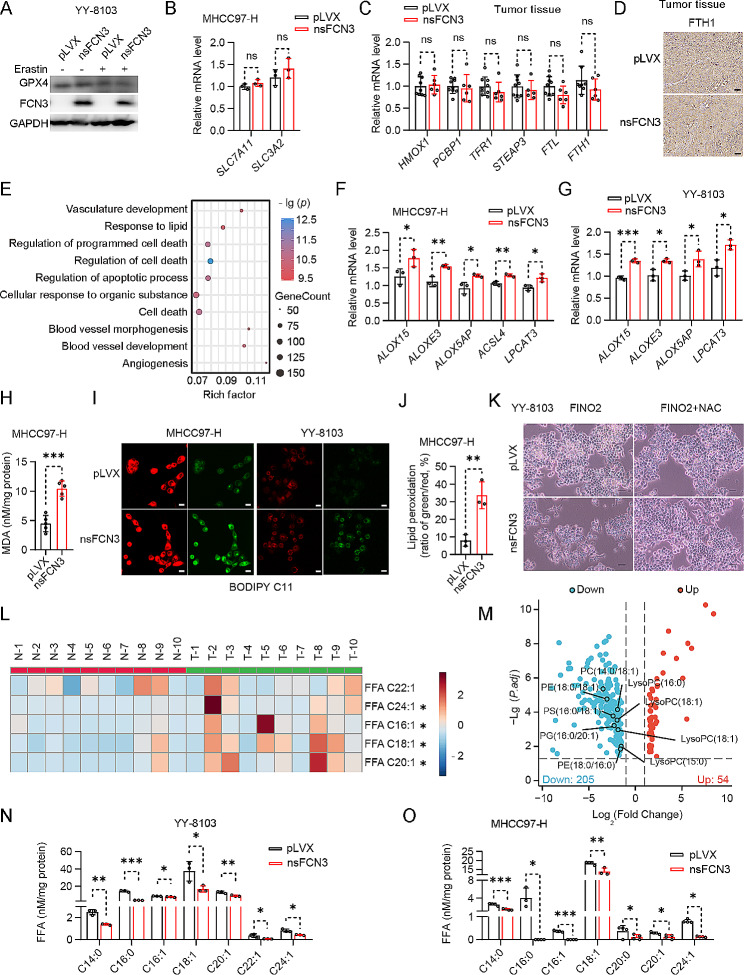
FCN3 promotes ferroptosis in HCC cells by reducing MUFA levels and enhancing lipid oxidation (**A**) Immunoblot of GPX4 in nsFCN3-overexpressed YY-8103 cells treated with 10 µM erastin for 24 h. GAPDH was used as a loading control. (**B**) mRNA levels of *SLC7A11* and *SLC3A2* in nsFCN3-overexpressed MHCC97-H cells and control cells. (**C**) mRNA levels of iron metabolism-related genes in xenograft tumors derived from Fig. 3H. (**D**) Representative images of FTH1 IHC staining in xenograft tumors derived from Fig. 3H. Scale bar, 40 μm. (**E**) The diagram of GO enrichment based on the DEGs (|log_2_ Fold Change|>1, *p* value < 0.05) in nsFCN3-overexpressed and control MHCC97-H cells analyzed with RNA-seq (*n* = 3). (**F**-**G**) mRNA levels of lipid peroxidation-related genes in nsFCN3-overexpressed MHCC97-H (**F**) cells and YY-8103 (**G**) cells. (**H**) The content of MDA in nsFCN3-overexpressed and control MHCC97-H cells (*n* = 5). (**I**-**J**) Representative images (**I**) and quantification (**J**) of BODIPY-C11 stained nsFCN3-overexpressed MHCC97-H and YY-8103 cells treated with 10 µM erastin for 12 h. A shift in green-to-red ratio indicates lipid oxidation, Scale bar, 40 μm. (**K**) Representative images of nsFCN3-overexpressed YY-8103 cells treated with FINO2 (10 µM) and NAC (1 mM). Scale bar, 100 μm. (**L**) Heatmap analysis of MUFA in tumor tissues (T) compared to paired normal adjacent tissue (N). Red indicated increase, and blue indicated decrease. -2 ~ 2 indicated the Fold Change. (**M**) Volcano plot of metabolites in nsFCN3-overexpressed and control MHCC97-H cells. LC-MS-based nontargeted metabolomic analysis, and the data was corrected by total peak area. (**N**-**O**) Fatty acid levels in YY-8103 (**N**) and MHCC97-H (**O**) cells overexpressing nsFCN3 Data are from one representative experiment of three independent experiments (F-G). Data are presented as mean ± SD. Significance was assessed by Student’s *t* test (B, C, F, G, H, J), Mann-Whitney *U* test (N, O). *p < 0.05, **p < 0.01, ***p < 0.001 compared with the control group. ns, not significant

We proceeded to investigate the mechanism by which FCN3 modulated ferroptosis. Firstly, we examined the impact of nsFCN3 overexpression on GPX4 protein levels. Intriguingly, we observed only marginal differences in GPX4 protein levels upon nsFCN3 expression, regardless of the presence and absence of erastin treatment (Fig. 4A). Moreover, the overexpression of nsFCN3 had minimal effects on the expression of two crucial cystine/glutamate transporters, *SLC7A11* and *SLC3A2* (Fig. 4B), both of which are known to be key regulators of GPX4 activation [30]. In addition, nsFCN3 overexpressing had no detectable effect on iron metabolism (Fig. 4C, D; Fig. S4A, B).

To gain a comprehensive understanding of how nsFCN3 promotes ferroptosis in HCC cells, we conducted RNA sequencing analysis of the cells overexpressing nsFCN3 (Fig. S4C). Our Gene Ontology (GO) analysis of biological processes revealed a significant alteration in the processes of lipid response and programmed cell death upon nsFCN3 overexpression (Fig. 4E). Given that lipid metabolism is closely related to ferroptosis [31], the GO analysis results suggested that FCN3 influenced ferroptosis by regulating lipid metabolism. Moreover, the RNA-seq analysis demonstrated the upregulation of key lipoxygenases (*ALOXE3* and *ALOX5AP*) upon nsFCN3 overexpression (Fig. S4D). Additionally, the overexpression of nsFCN3 significantly elevated the expression of the enzymes involved in lipid peroxidation (Fig. 4F, G).

Therefore, we further investigated the impact of FCN3 on the accumulation of malondialdehyde (MDA), the end product of lipid peroxidation in the context of ferroptosis. Our results demonstrated that nsFCN3 overexpression significantly elevated MDA levels, as compared to control cells (Fig. 4H). C11-BODIPY591/581 staining consistently indicated a markedly elevated percentage of lipid ROS in HCC cells overexpressing nsFCN3 (Fig. 4I, J; Fig. S4E). Next, we treated the HCC cells with FINO2 together with N-acetyl-L-cysteine (NAC), an ROS scavenger. Notably, we observed that NAC substantially attenuated the FINO2-induced ferroptosis in the cells overexpressing nsFCN3 (Fig. 4K). Collectively, these findings suggest that FCN3 exerts a significant influence on lipid peroxidation within the context of ferroptosis.

Given the pivotal role of MUFA in the inhibition of lipid peroxidation, we conducted a lipid metabolomics analysis with human HCC tumor specimens and their corresponding adjacent nontumor tissues. As shown in Fig. 4L and S4F, a variety of MUFA was upregulated in human HCC tissues compared to adjacent nontumor tissues. Conversely, untargeted metabolomics analysis also revealed a marked decrease in multiple free and membrane MUFA levels upon nsFCN3 overexpression (Fig. 4M). In line with this, targeted metabolomics approach further confirmed a substantial reduction in MUFA content in the cells overexpressing nsFCN3 (Fig. 4N, O).

Taken together, these results indicate that the overexpression of FCN3 decreases the content of MUFA, ultimately promoting ferroptosis in HCC cells.

### FCN3 reduces MUFA content in HCC through suppressing SREBP1c expression

**Fig. 5:**
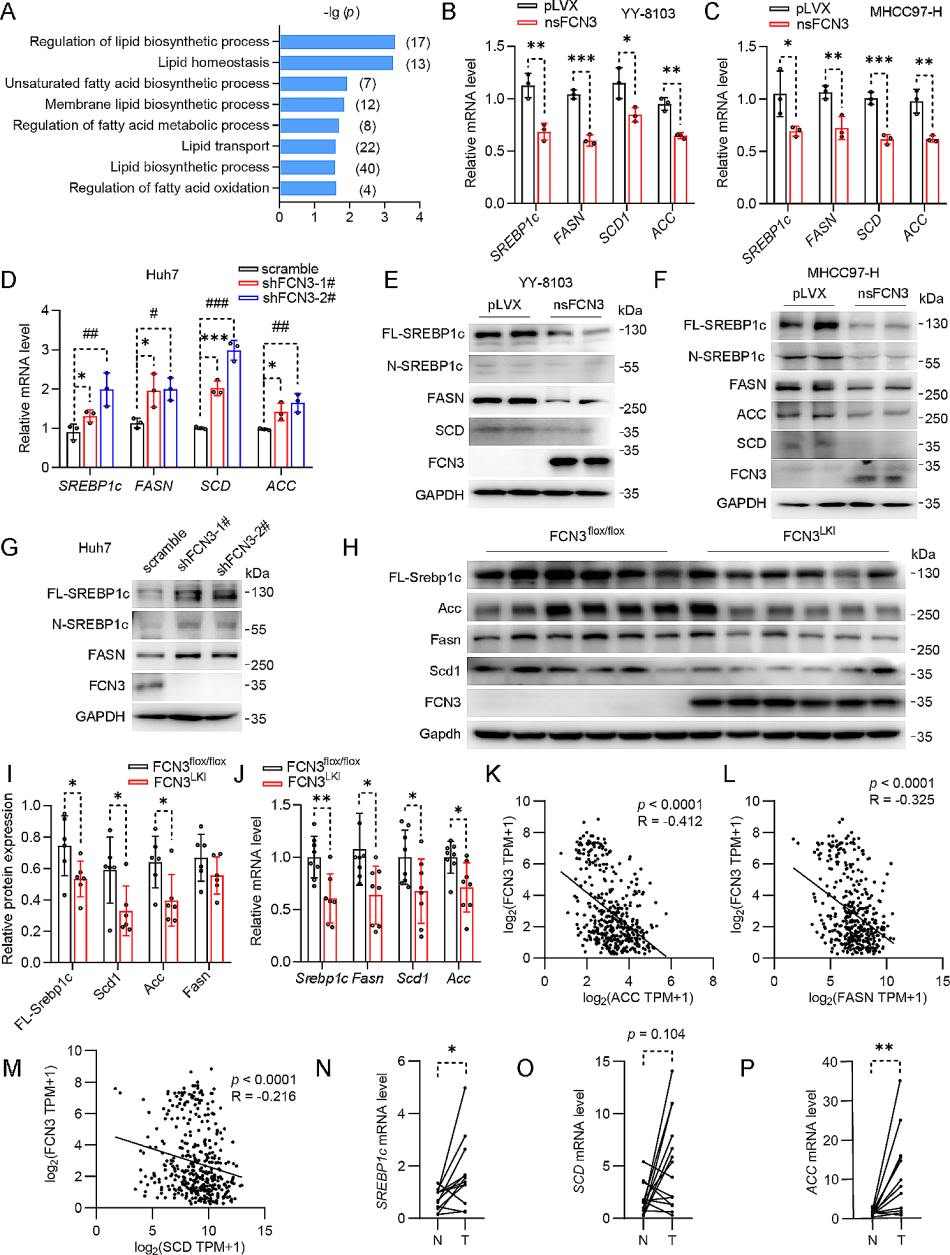
FCN3 inhibits MUFA synthesis by downregulating SREBP1c expression (**A**) Enrichment of lipid metabolism related biological processes based on the GO enrichment in Fig. 4E. (**B**-**C**) mRNA levels of lipogenic genes in nsFCN3-overexpressed YY-8103 (**B**) and MHCC97-H (**C**) cells. (**D**) mRNA levels of lipogenic genes in FCN3-knockdown and control Huh7 cells. (**E**-**F**) Immunoblots of lipogenic proteins in nsFCN3-overexpressed YY-8103 (**E**) and MHCC97-H (**F**) cells. GAPDH was used as a loading control. (**G**) Immunoblots of lipogenic proteins in FCN3-knockdown and control Huh7 cells. GAPDH was used as a loading control. (**H**-**I**) Immunoblots (**H**) and quantification (**I**) of lipogenic proteins in the liver tissues of FCN3^LKI^ and control mice. GAPDH was used as a loading control. (**J**) mRNA levels of lipogenic genes in the liver tissues of FCN3^LKI^ and control mice. (**K**-**M**) Correlation analysis of *ACC* (**K**), *FASN* (**L**), *SCD* (**M**) and *FCN3* expressions based on TCGA database. (**N**-**P**) mRNA levels of *SREBP1c* (**N**), *SCD* (**O**) and *ACC* (**P**) in 15 paired adjacent and tumor tissues. Data are from one representative experiment of three independent experiments (B-G). Data are presented as mean ± SD. Significance was assessed by Student’s *t* test (B, C, I, J), one-way ANOVA (D), Spearman correlation (K, L, M), paired Student’s *t* test (N, P), Wilcoxon matched-pairs signed rank test (O). *^, #^*p* < 0.05, **^, ##^*p* < 0.01, ***^, ###^*p* < 0.001 compared with the control group

To investigate the mechanism through which FCN3 reduces MUFA levels in HCC, we reevaluated the GO analysis results and found that a number of crucial lipid biosynthetic processes were enriched when nsFCN3 was overexpressed in HCC cells (Fig. 5A). In line with this, qPCR results revealed a significant decrease in the expression of *SREBP1c*, a key transcription factor responsible for upregulating the expression of lipid synthase, in HCC cells overexpressing nsFCN3 or FCN3 (Fig. 5B, C; Fig. S4G, H). We also observed a consistent reduction in the expression of the lipid synthases (*FASN* and *ACC*), and enzyme related to lipid desaturation (*SCD*) in cells overexpressing nsFCN3 or FCN3 (Fig. 5B, C; Fig. S4G, H). Conversely, the expression of *SREBP1c*, *FASN*, *SCD* and *ACC* increased in HCC cells with FCN3 knockdown (Fig. 5D). These observations were further validated through immunoblotting analysis (Fig. 5E-G; Fig. S4I, J). Moreover, we further detected a marked reduction in the expression of Srebp1c, Fasn, Scd1 and Acc in FCN3^LKI^ mice liver (Fig. 5H-J). Additionally, we also revealed that both FCN3 or nsFCN3 overexpression downregulated the intracellular TG levels across various HCC cell lines (Fig. S4K, L); while TG levels increased upon FCN3 knockdown (Fig. S4M). Furthermore, a correlation analysis using TCGA database revealed a negative correlation between FCN3 levels and the expression levels of lipid synthetase genes (Fig. 5K-M). Notably, the mRNA levels of *SREBP1* and other genes responsible for MUFA synthesis, were elevated in both HCC tissues and PVTT tissues (Fig. 5N-P; Fig. S4N-S).

In summary, our results indicate that FCN3 reduces intracellular MUFA levels by downregulating the expression of SREBP1c and lipid synthases.

### FCN3 induces ferroptosis by inhibiting the AKT/SREBP axis

**Fig. 6:**
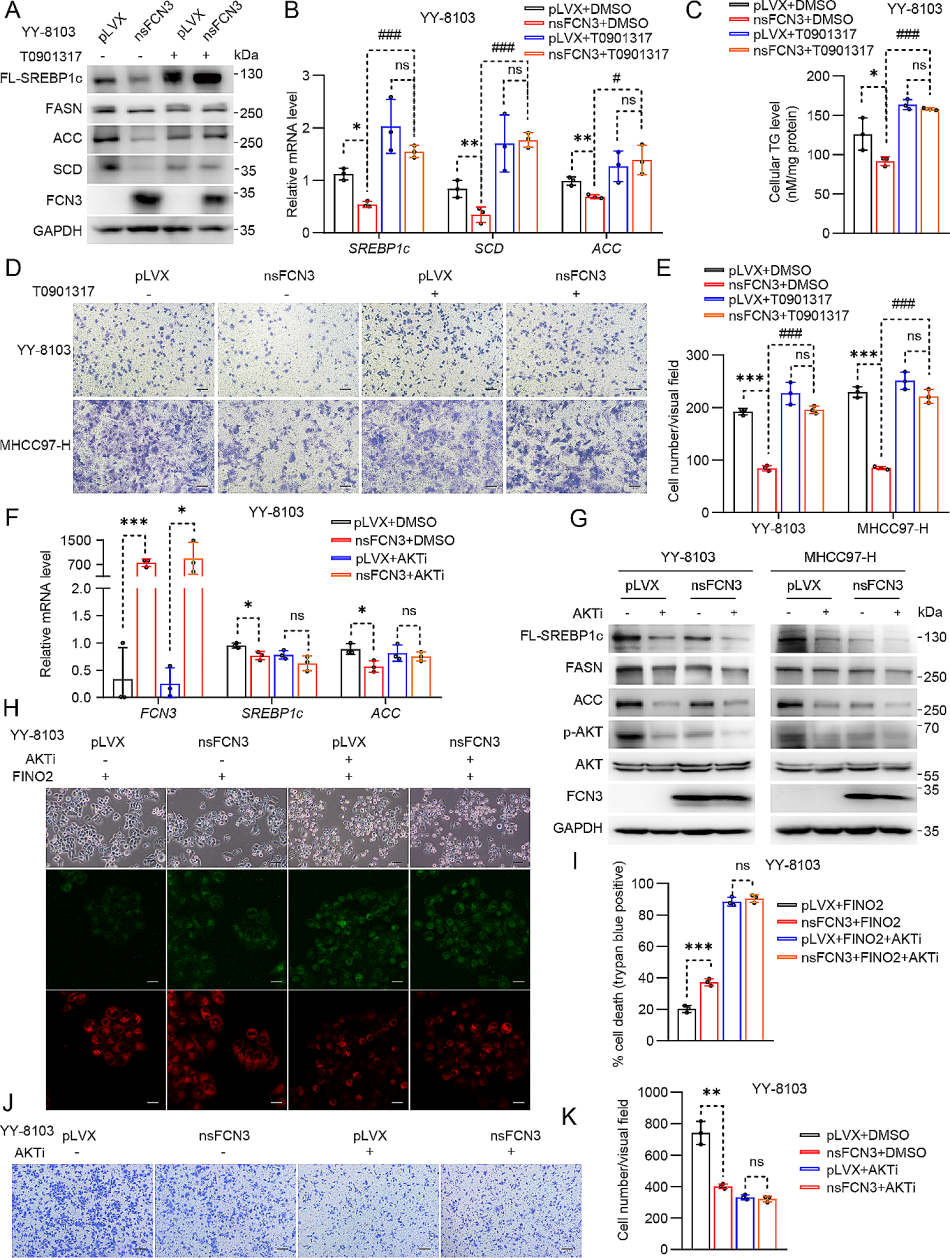
FCN3 suppresses HCC by inhibiting the AKT/SREBP axis (**A**) Immunoblots of DNL-related proteins in nsFCN3-overexpressed YY-8103 cells treated with T0901317 (10 µM) for 24 h. GAPDH was used as a loading control. (**B**) mRNA levels of lipogenic genes in nsFCN3-overexpressed and control YY-8103 cells treated with T0901317 (10 µM) for 24 h. (**C**) TG contents in nsFCN3-overexpressed YY-8103 cells treated with T0901317 (10 µM) and palmitic acid (PA, 100 µM). (**D**) Representative images of indicated cells that cultured in trans-well plates. Scale bar, 100 μm. (**E**) Quantification of the average number of migrating YY-8103 and MHCC97-H cells in (**D**). (**F**) mRNA levels of lipogenic genes in nsFCN3-overexpressed YY-8103 cells treated with AKTi (10 µM) for 24 h. (**G**) Immunoblots of lipogenic proteins and p-AKT in nsFCN3-overexpressed YY-8103 and MHCC97-H cells treated with AKTi (10 µM) for 24 h. GAPDH was used as a loading control. (**H**) Representative images of nsFCN3-overexpressed YY-8103 cells treated with FINO2 (5 µM) and AKTi (10 µM). Scale bar, 100 μm. (**I**) Quantification of trypan blue staining for death cell in (**H**). (**J**) Representative images of indicated cells that cultured in trans-well plates. Scale bar, 100 μm. (**K**) Quantification of the average number of migrating YY-8103 cells in (**J**) Data are presented as mean ± SD and are from one representative experiment of three independent experiments. Significance was assessed by Student’s *t* test (B, C, E, F, I, K). *^, #^*p* < 0.05, **^, ##^*p* < 0.01, ***^, ###^*p* < 0.001 compared with the control group. ns, not significant

Next, we aimed to verify the significance of SREBP1c in the inhibitory effects of FCN3 on HCC progression. Given the unavailability of a commercial SREBP1c agonist and the well-documented ability of LXRα to directly activate the transcription of *SREBP1c* [32], we conducted experiments involving cells overexpressing nsFCN3, as well as control cells, treated with T0901317, an LXRα agonist, to induce SREBP1c activation. The results demonstrated that T0901317 effectively abolished nsFCN3-mediated suppression in the expression of *SREBP1c* and other lipid synthases (Fig. 6A, B; Fig. S5A, B). Moreover, T0901317 also blunted the nsFCN3-mediated reduction in intracellular TG abundance (Fig. 6C; Fig. S5C). These results further confirmed that FCN3 diminished intracellular lipid synthesis in a SREBP1c-dependent manner. Notably, trans-well assays demonstrated that T0901317 impaired the ability of nsFCN3 to inhibit the migration of HCC cells (Fig. 6D, E).

Fatty acid oxidation (FAO) and lipid absorption are two other pivotal processes in lipid metabolism [33]. We observed that nsFCN3 exerted a negligible impact on the expression of FAO-associated genes (*CPT1α*, *PPARα* and *ACOX1*) (Fig. S5D-F), as well as genes related to lipid transport (*MTTP*, *FATP1* and *CD36*) (Fig. S5G-I). Additionally, a correlation analysis using the TCGA database revealed indistinctive correlations between FCN3 and FAO genes (Fig. S5J-L), as well as absence of notable correlations between FCN3 and lipid transport genes (Fig. S5M, N).

Taken together, these results suggest that FCN3 promotes HCC ferroptosis by specifically inhibiting MUFA synthesis through a SREBP1c-dependent mechanism.

### FCN3 suppresses lipid synthesis by inhibiting the IR/AKT signaling pathway

To elucidate the intricate mechanism underlying FCN3-mediated regulation of lipid synthesis, we conducted comprehensive Kyoto Encyclopedia of Genes and Genomes (KEGG) pathway enrichment analysis utilizing RNA sequencing data from Fig. 4E. Notably, our analysis revealed that the PI3K-AKT signaling pathway was one of the most significantly enriched pathways (Fig. S6A). Previous reports indicated that activated AKT promoted the process of lipid synthesis [34]. In line with this, overexpression of nsFCN3 led to a decrease in the phosphorylation levels of AKT (Fig. S6B, C). Moreover, the HCC tumors overexpressing nsFCN3 exhibited diminished p-AKT levels compared to control tumors (Fig. S6D, E). In addition, we assessed the levels of p-AKT (S473) and AKT in 18 pairs of HCC samples and their corresponding adjacent normal tissues and found elevated AKT phosphorylation levels in HCC samples (T) in contrast to control tissues (N) (Fig. S6F, G). Subsequently, we employed AKTi, an AKT-specific inhibitor, to treat HCC cells overexpressing nsFCN3 and control cells. The results demonstrated that AKTi abolished the downregulation of lipid synthases caused by nsFCN3 overexpression (Fig. 6F, G; Fig. S6H). Additionally, AKTi treatment abrogated the disparity in cell sensitivity to ferroptosis and in the content of lipid ROS between nsFCN3-overexpressing cells and control cells (Fig. 6H, I; Fig. S6I). Consequently, the inhibitory effect of nsFCN3 on HCC cell migration was also attenuated by AKTi treatment (Fig. 6J, K). Intriguingly, statistical analysis unveiled an inverse correlation between FCN3 expression level and the ratio of p-AKT (S473)/AKT (Fig. S6J). Taken together, these results indicate that FCN3 regulates lipid synthesis and ferroptosis by inhibiting AKT activation.

**Fig. 7:**
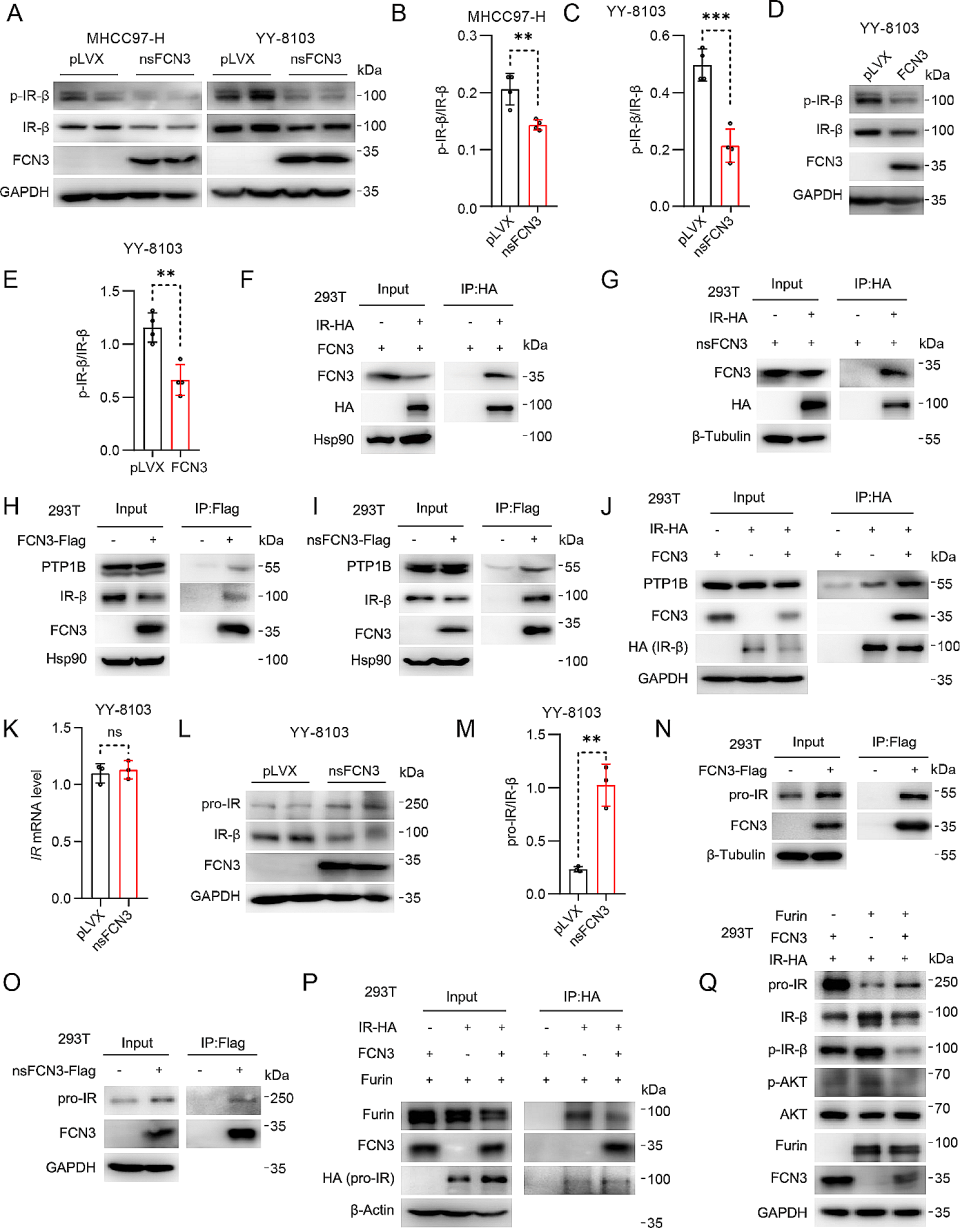
FCN3 inhibits IR activation by promoting the dephosphorylation of IR-β and suppressing pro-IR cleavage (**A**) Immunoblots of p-IR-β and IR-β in nsFCN3-overexpressed MHCC97-H and YY-8103 cells. GAPDH was used as a loading control. (**B**-**C**) Quantitative analysis of p-IR-β/IR-β ratio in nsFCN3-overexpressed MHCC97-H (**B**) and YY-8103 (**C**) cells. (**D**-**E**) Immunoblots (**D**) and quantification (**E**) of p-IR-β and IR-β in FCN3-overexpressed YY-8103 cells. GAPDH was used as a loading control. (**F**-**G**) Immunoblots of FCN3 and HA-tagged IR after immunoprecipitation of HA-tagged IR from HEK-293T cells that transfected with FCN3 (**F**) or nsFCN3 (**G**) and IR-HA. (**H**-**I**) Immunoblots of IR-β, PTP1B and FCN3 after immunoprecipitation of Flag-tagged FCN3 from HEK-293T cells that transfected with FCN3-Flag (**H**) or nsFCN3-Flag (**I**). (**J**) Immunoblots of PTP1B, FCN3 and HA-tagged IR after immunoprecipitation of HA-tagged IR from indicated HEK-293T cells. (**K**) mRNA level of IR in nsFCN3-overexpressed YY-8103 cells. (**L**-**M**) Immunoblots (**L**) and quantification (**M**) of pro-IR and IR-β in nsFCN3-overexpressed YY-8103 cells. GAPDH was used as a loading control. (**N**-**O**) Immunoblots of pro-IR and FCN3 after immunoprecipitation of Flag-tagged FCN3 from HEK-293T cells that transfected with FCN3-Flag (**N**) or nsFCN3-Flag (**O**). (**P**) Immunoblots of Furin, FCN3 and HA-tagged pro-IR after immunoprecipitation of HA-tagged IR from indicated HEK-293T cells. (**Q**) Immunoblots of indicated proteins in HEK-293T cells. GAPDH was used as a loading control Data are from one representative experiment of three independent experiments (A-E and K-M). Data are presented as mean ± SD. Significance was assessed by Student’s *t* test (B, C, E, K, M). **p < 0.01, ***p < 0.001 compared with the control group. ns, not significant

To unravel the underlying mechanisms behind FCN3-mediated AKT inhibition, we explored the activation of IR signaling pathway upstream of AKT. The results illuminated that overexpression of both FCN3 and nsFCN3 significantly decreased the levels of phosphorylated IR-β (Fig. 7A-E), while the expression of pivotal phosphatases within the IR-AKT signaling pathway remained unaltered (Fig. S6K, L). Conversely, recombinant FCN3 exhibited minimal effect on the phosphorylation levels of IR and AKT (Fig. S6M). To explore the mechanism by which FCN3 downregulates IR-β phosphorylation, we performed a coimmunoprecipitation assay with exogenously overexpressed IR and FCN3 or nsFCN3 and found that both FCN3 and nsFCN3 interacted strongly with IR (Fig. 7F, G). Furthermore, in FCN3-Flag- or nsFCN3-Flag-overexpressing HEK-293T cells, a reverse semi-endogenous coimmunoprecipitation assay with a Flag antibody revealed a strong interaction between FCN3 or nsFCN3 and endogenous IR-β (Fig. 7H, I). Intriguingly, we also detected the binding of both FCN3 and nsFCN3 to the protein tyrosine phosphatase nonreceptor type 1 (PTP1B), a phosphatase responsible for dephosphorylation of IR-β [35] (Fig. 7H, I). Notably, we demonstrated that FCN3 overexpression substantially enhanced the interaction between IR-β and PTP1B (Fig. 7J). Collectively, these findings indicate that FCN3 inhibits the phosphorylation and subsequent activation of IR-β via PTP1B.

In the above experiment, we observed that cells overexpressing FCN3 and nsFCN3 exhibited a reduction in the total protein levels of IR-β (Fig. 7A, D, H), while the mRNA level of IR remained unaltered (Fig. 7K). Given the process of generating IR-α and IR-β through pro-insulin receptor (pro-IR) cleavage [36], we investigated the impact of FCN3 on pro-IR levels. Notably, overexpression of nsFCN3 resulted in an obvious increase in pro-IR protein levels while reducing IR-β abundance (Fig. 7L, M), suggesting a potential influence of FCN3 on the pro-IR cleavage process. Moreover, immunoprecipitation assays revealed robust interactions between both FCN3 and nsFCN3 with pro-IR (Fig. 7N, O). Given that Furin is reported as a cleavage enzyme targeting pro-IR [36], we investigated the interaction between pro-IR and Furin in the presence and absence of FCN3. The results revealed that FCN3 overexpression impeded the pro-IR/Furin interaction (Fig. 7P). Notably, by co-overexpressing IR-β, Furin, and FCN3, we found that the overexpression of Furin led to a reduction in pro-IR levels, accompanied by an increase in IR-β levels (Fig. 7Q). Furthermore, the additional overexpression of FCN3 resulted in elevated pro-IR levels and diminished IR-β levels compared to the group with only Furin overexpression (Fig. 7Q). Moreover, we found that the overexpression of FCN3 also suppressed the phosphorylation of IR-β and AKT in the presence of Furin overexpression (Fig. 7Q). These collective findings indicate that FCN3 suppresses Furin-mediated pro-IR cleavage, thereby impeding the generation of IR-β.

In conclusion, our results highlight that FCN3 exerts inhibitory effects on IR-β phosphorylation by enhancing PTP1B-mediated dephosphorylation of IR-β, while concurrently attenuating Furin-driven pro-IR splicing, thereby intricately regulating IR-β and AKT activation.

## Discussion

The development of cancer is closely linked to ferroptosis [7]. It is theoretically possible that cancer cells are more vulnerable to ferroptosis than normal cells since they require more iron and lipids than normal cells for proliferation. Even if the tumor is resistant to traditional drug-induced apoptosis, it may remain highly sensitive to ferroptosis inducers [3]. Targeting ferroptosis has therefore been recognized as a promising approach for cancer treatment [37]. Sorafenib, an FDA-approved drug for the treatment of liver, renal, and thyroid carcinoma, has been found to induce ferroptosis in tumors by inhibiting glutathione biosynthesis [38]. Although this drug has demonstrated some efficacy in treating HCC, clinical studies have shown that a subset of patients do not respond to sorafenib, leading to disease progression. Therefore, investigating the mechanisms underlying resistance to ferroptosis in HCC is of critical significance. In our study, we reveal an unexpected role of FCN3, a complement system component, in reversing ferroptosis resistance in HCC cells.

One well-established cause of ferroptosis resistance is genetic mutations and epigenetic changes in cancer cells [39], which lead to abnormal function of these genes, including phosphatidylinositol 3-kinase (*PI3K*), serine/threonine kinase 11 (*STK11*), Kelch-like ECH-associated protein 1 (*KEAP1*), and *SREBP2* etc. Activating mutation of PI3K, a highly frequent event in human cancer, confers ferroptosis resistance by hyperactivation of mTOR-SREBP1-SCD1 signaling in breast cancer cells [40]. Concurrent loss-of-function mutations in STK11 and KEAP1 in lung adenocarcinoma result in significantly elevated expression of ferroptosis-protective genes, such as AKR1C1/2/3, and resistance to pharmacologically induced ferroptosis [41]. Aberrantly expressed SREBP2 directly induces the transcription of the iron carrier transferrin, resulting in reduced intracellular iron pools, decreased ROS levels, and suppressed lipid peroxidation, thus conferring resistance to ferroptosis inducer [42].

Lipid metabolism is closely related to cancer progression. First, lipids promote tumor development and metastasis by supplying energy. For instance, mutant KRAS upregulates long-chain-fatty-acid–CoA ligase (ACSL) expression, thereby increasing β-oxidation and ATP production to support lung cancer development [43]. In triple-negative breast cancer, the oncogenic transcription factor MYC is abnormally increased, activating cellular FAO to promote cell proliferation [44]. Second, reprogramming of lipid metabolism in tumor cells often leads to ferroptosis resistance. Specifically, some tumors develop resistance to ferroptosis by reducing PUFA-ePL levels [8]. Conversely, intracellular PUFA accumulation has been shown to induce ferroptosis and suppress prostate tumor cell proliferation and migration [45]. On the other hand, SCD1-mediated MUFA biosynthesis decreases cell susceptibility to RSL3-induced ferroptosis, leading to increased colorectal cancer cell proliferation, migration and invasion [46]. SCD1 is upregulated in ovarian cancer, which consequently leads to ferroptosis resistance. Pharmacological inhibition of SCD1 enhances the antitumor efficacy of ferroptosis inducers [18]. Furthermore, it has been reported that cancer cells grown in vitro under lipid-depleted conditions undergo ER stress and cell death upon SCD1 inhibition [47].

**Fig. 8:**
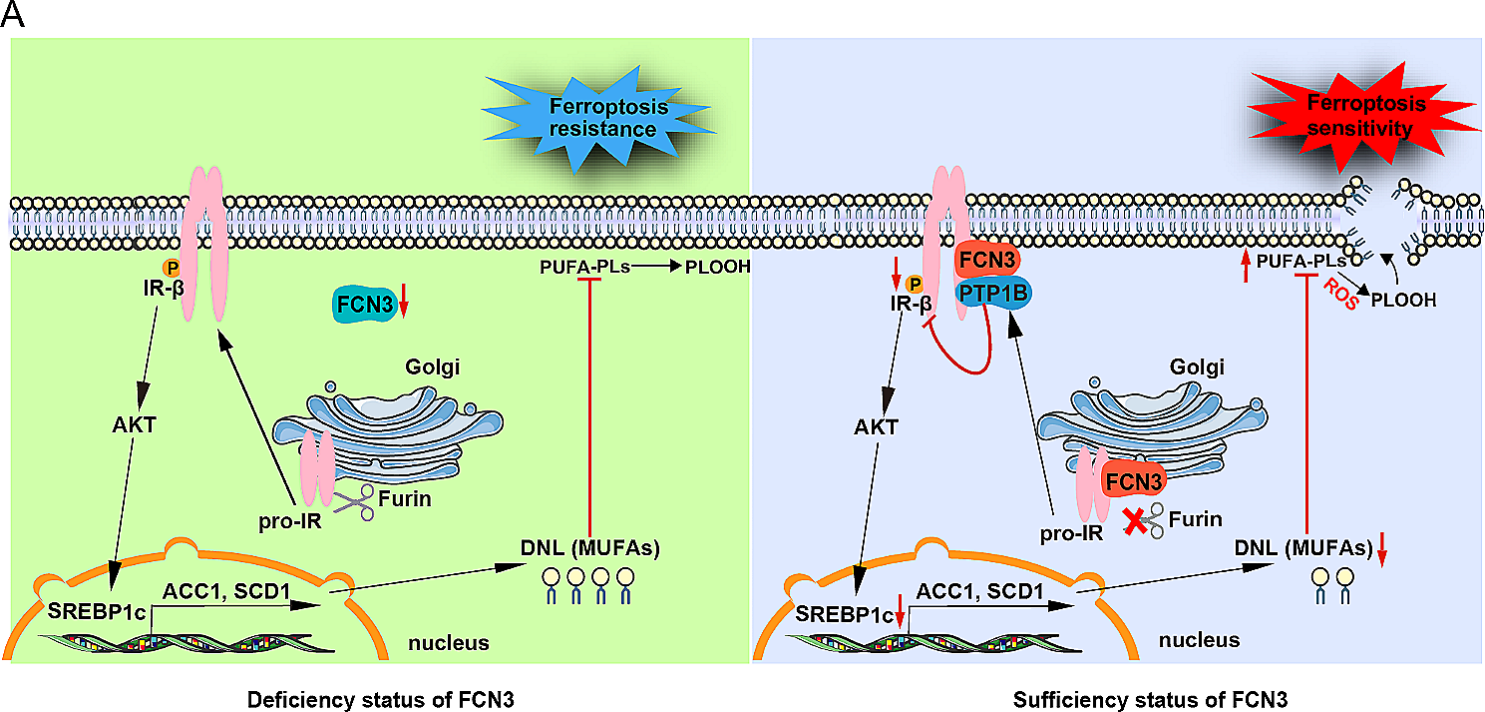
Schematic summary displays that FCN3 deficiency in HCC causes the overactivation of IR-β and AKT, which further promotes the expression of the downstream transcription factor SREBP1c. Then, the expression of DNL and lipid desaturation related genes including ACC1, FASN and SCD1 are upregulated, resulting in increased intracellular MUFA, which promotes a ferroptosis-resistant cell state. In contrast, overexpression of FCN3 enhances lipid peroxidation through inhibiting IR-AKT-DNL axis and thereby sensitizes HCC cells to ferroptosis

In our study, we revealed that overexpression of FCN3 could significantly enhance the sensitivity of HCC cells to ferroptosis, leading to reduced survival and migration. Mechanistically, we demonstrated that FCN3 inhibits the activation of IR-β by reducing both its phosphorylation and the cleavage of its pro-form, resulting in decreased phosphorylation of IR-β and AKT, as well as the expression of SREBP1c and downstream genes regulating DNL and fatty acid desaturation, such as ACC1, FASN and SCD1. Consequently, FCN3 diminished intracellular MUFA and sensitized HCC cells to ferroptosis (Fig. 8). Besides, analysis of clinical samples showed that FCN3 was significantly downregulated in HCC, and FCN3 expression was negatively associated with the progression of HCC. In line with this, MUFA levels in HCC were increased compared to those in corresponding nontumor tissues. Collectively, our findings broaden the comprehension of the complement system factors’ novel roles in tumor metabolic reprogramming and the regulation of ferroptosis. Furthermore, they highlight the potential of targeting FCN3-induced ferroptosis as an effective strategy for HCC treatment.

Emerging investigations have pointed to a state of excessive IR signaling activation in HCC, resulting in the subsequent overactivation of AKT [48]. However, the precise regulatory mechanism underlying this phenomenon remains elusive. In our study, we revealed that FCN3 interacts with pro-IR and suppresses its cleavage, resulting in a reduction in IR-β levels. Moreover, FCN3 and IR-β may interact with each other in the intracellular membrane, recruiting PTP1B to dephosphorylate IR-β. Therefore, the significant reduction in FCN3 levels in HCC may cause overactivation of IR and AKT.

Increasing research has revealed that complement system components play a crucial role in the progression of cancer. For example, FCN2 inhibits the metastasis of HCC by regulating the TGF-β/Smad signaling pathway [21]. FCN3 has been previously reported to facilitate apoptosis and suppress cell proliferation by upregulating the expression of p53 [23]. p53 plays a crucial role as a tumor suppressor gene in cancer, not only inhibiting the cell cycle, promoting senescence and apoptosis but also enhancing the sensitivity of tumor cells to ferroptosis [49]. Furthermore, it has been documented that AKT can bind directly to MDM4, inhibiting its protein degradation, while MDM4 promotes the degradation of p53. Hence, the activation of AKT could reduce p53 protein levels. In our study, we discovered that FCN3 enhanced the sensitivity of HCC cells to ferroptosis by inhibiting the phosphorylation of IR and AKT [50]. The upregulation of p53 expression may serve as a downstream signaling of reduced phosphorylation level of AKT and promotes the sensitivity of HCC cell to ferroptosis. Thus, the enhancement of p53 expression by FCN3, as identified in prior research, aligns with and supports the mechanisms and phenotypic observations detailed in our study.

## Conclusions

In this study, we reveal that a significant decrease in FCN3 expression within human HCC specimens correlates positively with HCC cells ferroptosis resistance. We demonstrate that FCN3 overexpression efficiently sensitizes HCC cells to ferroptosis by downregulating MUFA levels, leading to the inhibition of the oncogenesis and progression of both primary hepatocellular carcinoma and subcutaneous HCC xenograft. Notably, we discover that FCN3 directly binds to the IR-β and pro-IR, inhibiting pro-IR cleavage and IR-β phosphorylation, ultimately resulting in suppressing of the IR-β/SREBP1c axis-mediated MUFA synthesis. Taken together, our study not only expands comprehensive understanding of ferroptosis sensitivity regulation in HCC involving FCN3, but also provides a promising strategy for HCC treatment.

### Electronic supplementary material

Below is the link to the electronic supplementary material.

Supplementary Material 1

Supplementary Material 2

Supplementary Material 3

Supplementary Material 4

Supplementary Material 5

## Data Availability

All data associated with this study are present in the paper or the supplementary materials. The datasets generated by RNA sequencing are available from Mendeley data (10.17632/z6hky6jzzr.1). Any additional information required to reanalyze the data reported in this paper is available from the lead contact upon request.

## Abbreviations

HCC: hepatocellular carcinoma
SREBP1c: sterol regulatory element binding protein-1c
DNL: *de novo* lipogenesis
ACC: acetyl-CoA carboxylase
FASN: fatty acid synthase
SCD1: stearoyl-CoA desaturase 1
MUFAs: monounsaturated fatty acids
GSH: glutathione
GPX4: glutathione peroxidase 4
PUFAs: polyunsaturated fatty acids
PUFA-PLs: PUFA-containing phospholipids
ROS: reactive oxygen species
PUFA-ePLs: polyunsaturated ether phospholipids
FCN: Ficolin
PVTT: portal vein tumor thrombus
TCGA: The cancer genome atlas
EMT: epithelial-mesenchymal transition
DEGs: differentially expressed genes
GO: Gene Ontology
MDA: malondialdehyde
SFAs: saturated fatty acids
TG: Triglyceride
FAO: fatty acid oxidation
CPT1α: carnitine palmitoyltransferase 1A
PPARα: peroxisome proliferator activated receptor alpha
ACOX1: acyl-CoA oxidase 1
MTTP: microsomal triglyceride transfer protein
FATP1: fatty acid transport protein 1
DGAT1/2: diacylglycerol O-acyltransferase 1/2
GPAT: glycerol-3-phosphate acyltransferase
KEGG: kyoto encyclopedia of genes and genomes
PI3K: phosphatidylinositol 3-kinase
STK11: serine/threonine kinase 11
KEAP1: kelch-like ECH-associated protein 1
DMEM: Dulbecco’s modified Eagle medium
RNA-seq: RNA sequencing
RPMI: Roswell Park Memorial Institute
AKT: protein kinase B
mTOR: mammalian target of rapamycin
PBS: phosphate buffered saline
IR: insulin receptor
PTP1B: protein tyrosine phosphatase non-receptor type 1
Furin: paired basic amino acid cleaving enzyme
PTGS2: prostaglandin-endoperoxide synthase 2
TMA: tissue microarray
ACSL4: acyl-CoA synthetase long-chain family member 4
NRF2: nuclear factor erythroid 2-like 2
FFA: free fatty acid
HMOX1: heme oxygenase 1
FTH1: ferritin heavy chain 1
FTL: ferritin light chain
PCBP1: poly(RC) binding protein 1
TFR1: transferrin receptor
STEAP3: signal transducer and activator of transcription 3
CCl_4_: carbon tetrachloride
