# Supplementary material for: Ficolin 3 promotes ferroptosis in HCC by downregulating IR/SREBP axis-mediated MUFA synthesis
Source: J Exp Clin Cancer Res. 2024 May 3;43:133. doi: 10.1186/s13046-024-03047-2 (PMC11067213; doi:10.1186/s13046-024-03047-2)
Supplement: Supplementary file 2 — Supplementary Material 2 [file 13046_2024_3047_MOESM2_ESM.docx]

**Table S4. The expression of complement member in HCC and their association with HCC progression.**

| genes | HR (High) | *p* (HR) | Fold change (log_2_ [T/N]) |
| --- | --- | --- | --- |
| CRP | 1 | 0.81 | -4.481 |
| FCN3 | 0.68 | 0.033 | -4.456 |
| C9 | 0.93 | 0.68 | -4.302 |
| C7 | 0.68 | 0.03 | -2.788 |
| C6 | 0.67 | 0.026 | -2.082 |
| MBL2 | 0.96 | 0.81 | -1.932 |
| C8A | 0.72 | 0.066 | -1.802 |
| C1R | 0.87 | 0.42 | -1.394 |
| C8B | 0.54 | 0.00065 | -1.352 |
| MASP1 | 0.78 | 0.16 | -1.202 |
| C1RL | 0.66 | 0.022 | -1.050 |
| C1S | 0.84 | 0.33 | -0.935 |
| C4BPA | 0.73 | 0.082 | -0.922 |
| MASP2 | 0.69 | 0.041 | -0.743 |
| C1QB | 1.1 | 0.75 | -0.655 |
| C1QA | 1.1 | 0.59 | -0.529 |
| C3 | 0.82 | 0.26 | -0.506 |
| CFP | 0.76 | 0.12 | -0.484 |
| CFD | 1.2 | 0.24 | -0.482 |
| C5 | 0.81 | 0.24 | -0.469 |
| FCN1 | 0.96 | 0.83 | -0.386 |
| C5AR1 | 1.3 | 0.13 | -0.385 |
| C4B | 0.85 | 0.36 | -0.359 |
| CR1 | 1.1 | 0.7 | -0.349 |
| C4A | 0.84 | 0.31 | -0.285 |
| CFH | 0.77 | 0.15 | -0.204 |
| C3AR1 | 1.2 | 0.26 | -0.027 |
| C8G | 0.89 | 0.49 | 0.138 |
| C4BPB | 0.86 | 0.39 | 0.165 |
| CFB | 0.66 | 0.02 | 0.185 |
| C2 | 0.84 | 0.34 | 0.307 |
| C5AR2 | 1.4 | 0.082 | 0.648 |

log_2_(T/N) value < 0 indicated that gene expression was downregulated, while log_2_(T/N) value > 0 indicated that gene expression was upregulated in HCC; *p* (HR) < 0.05 values are set for highly significant differences; HR > 1 (indicating an unfavorable factor for prognosis) and HR < 1 (indicating a protective factor for prognosis).
