# Supplementary material for: Ficolin 3 promotes ferroptosis in HCC by downregulating IR/SREBP axis-mediated MUFA synthesis
Source: J Exp Clin Cancer Res. 2024 May 3;43:133. doi: 10.1186/s13046-024-03047-2 (PMC11067213; doi:10.1186/s13046-024-03047-2)
Supplement: Supplementary file 4 — Supplementary Material 4 [file 13046_2024_3047_MOESM4_ESM.docx]

**Table S3. Primers used for real-time PCR and shRNA construction.**

| **real-time PCR primers** | | |  |  |
| --- | --- | --- | --- | --- |
| NO. | Primers | Sequences | Product length | Sources |
| 1 | 18S F (human) | 5’-TTCGAACGTCTGCCCTATCAA-3’ | 50 | This paper |
|  | 18S R (human) | 5’-ATGGTAGGCACGGCGACTA-3’ |  |  |
| 2 | actin F (human) | 5’-CATGTACGTTGCTATCCAGGC-3’ | 250 | This paper |
|  | actin R (human) | 5’-CTCCTTAATGTCACGCACGAT-3’ |  |  |
| 3 | FCN3 F (human) | 5’-CTGGCACTGGGCAAGTTCTC-3’ | 93 | This paper |
|  | FCN3 R (human) | 5’-ATCGTGGTCAGCGTCATAGGT-3’ |  |  |
| 4 | PTGS2 F (human) | 5’-TAAGTGCGATTGTACCCGGAC-3’ | 250 | This paper |
|  | PTGS2 R (human) | 5’-TTTGTAGCCATAGTCAGCATTGT-3’ |  |  |
| 5 | SREBF1 F (human) | 5’-TGAGGACAGCAAGGCAAAGC-3’ | 109 | This paper |
|  | SREBF1 R (human) | 5’-GCAGGACAGGCAGAGGAAGA-3’ |  |  |
| 6 | ACC F (human) | 5’-ACCTGCGAGTAGAGACACAATTC-3’ | 130 | This paper |
|  | ACC R (human) | 5’-TTCTTGGTGACTTGAGCGTGAG-3’ |  |  |
| 7 | FASN F (human) | 5’-ACAGCGGGGAATGGGTACT-3’ | 188 | This paper |
|  | FASN R (human) | 5’-GACTGGTACAACGAGCGGAT-3’ |  |  |
| 8 | SCD F (human) | 5’-TGGCTTGCTGATGATGTGCTT-3’ | 251 | This paper |
|  | SCD R (human) | 5’-AGGAGTGGTGGTAGTTGTGGAA-3’ |  |  |
| 9 | PPARα F (human) | 5’-AGAGAGCCCGTTATCTGAAGAGT-3’ | 200 | This paper |
|  | PPARα R (human) | 5’-CCACAGGATAAGTCACCGAGGA-3’ |  |  |
| 10 | CPT1α F (human) | 5’-TCACATTCAGGCAGCAAGAGC-3’ | 126 | This paper |
|  | CPT1α R (human) | 5’-TGAGCGGAGCAGAGTGGAAT-3’ |  |  |
| 11 | ACOX1 F (human) | 5’-GGAACTCACCTTCGAGGCTTG-3’ | 164 | This paper |
|  | ACOX1 R (human) | 5’-TTCCCCTTAGTGATGAGCTGG-3’ |  |  |
| 12 | CD36 F (human) | 5’-AAGCCAGGTATTGCAGTTCTTT-3’ | 220 | This paper |
|  | CD36 R (human) | 5’-GCATTTGCTGATGTCTAGCACA-3’ |  |  |
| 13 | MTTP F (human) | 5’-ACAAGCTCACGTACTCCACTG-3’ | 111 | This paper |
|  | MTTP R (human) | 5’-TCCTCCATAGTAAGGCCACATC-3’ |  |  |
| 14 | FATP1 F (human) | 5’-GGGGCAGTGTCTCATCTATGG-3’ | 111 | This paper |
|  | FATP1 R (human) | 5’-CCGATGTACTGAACCACCGT-3’ |  |  |
| 15 | INSR F (human) | 5’-AAAACGAGGCCCGAAGATTTC-3’ | 90 | This paper |
|  | INSR R (human) | 5’-GAGCCCATAGACCCGGAAG-3’ |  |  |
| 16 | ALOX5AP F (human) | 5’-AAACGCATCATACTCTTCCTGTT-3’ | 138 | This paper |
|  | ALOX5AP R (human) | 5’-GGGAATGAGAAGTAGAGGGGAG-3’ |  |  |
| 17 | ACSL4 F (human) | 5’-ACTGGCCGACCTAAGGGAG-3’ | 122 | This paper |
|  | ACSL4 R (human) | 5’-GCCAAAGGCAAGTAGCCAATA-3’ |  |  |
| 18 | LPCAT3 F (human) | 5’-GGAGACCTACCTCATCCACCT-3’ | 132 | This paper |
|  | LPCAT3 R (human) | 5’-CGGCCCATTAGTCGAAGGA-3’ |  |  |
| 19 | ALOX15 F (human) | 5’-GGGCAAGGAGACAGAACTCAA-3’ | 168 | This paper |
|  | ALOX15 R (human) | 5’-CAGCGGTAACAAGGGAACCT-3’ |  |  |
| 20 | ALOXE3 F (human) | 5’-TGTCACCGAACCGGATGGTA-3’ | 171 | This paper |
|  | ALOXE3 R (human) | 5’-CGGTAGCATTCTTGTCGGG-3’ |  |  |
| 21 | SLC7A11 F (human) | 5’-TCTCCAAAGGAGGTTACCTGC-3’ | 123 | This paper |
|  | SLC7A11 R (human) | 5’-AGACTCCCCTCAGTAAAGTGAC-3’ |  |  |
| 22 | SLC3A2 F (human) | 5’-CTGGTGCCGTGGTCATAATC-3’ | 164 | This paper |
|  | SLC3A2 R (human) | 5’-GCTCAGGTAATCGAGACGCC-3’ |  |  |
| 23 | HMOX1 F (human) | 5’-AAGACTGCGTTCCTGCTCAAC-3’ | 247 | This paper |
|  | HMOX1 R (human) | 5’-AAAGCCCTACAGCAACTGTCG-3’ |  |  |
| 24 | STEAP3 F (human) | 5’-CTCCCCGGAGGTCATCTTTG-3’ | 117 | This paper |
|  | STEAP3 R (human) | 5’-TCTTGCTCTGTAGGGTTGCTC-3’ |  |  |
| 25 | FTL F (human) | 5’-CAGCCTGGTCAATTTGTACCT-3’ | 114 | This paper |
|  | FTL R (human) | 5’-GCCAATTCGCGGAAGAAGTG-3’ |  |  |
| 26 | TFR1 F (human) | 5’-ACCATTGTCATATACCCGGTTCA-3’ | 219 | This paper |
|  | TFR1 R (human) | 5’-CAATAGCCCAAGTAGCCAATCAT-3’ |  |  |
| 27 | PCBP1 F (human) | 5’-AAAGGCGGGTGTAAGATCAAAG-3’ | 148 | This paper |
|  | PCBP1 R (human) | 5’-GGCAAATCTGCTTGACACACTC-3’ |  |  |
| 28 | NRF2 F (human) | 5’-TCAGCGACGGAAAGAGTATGA-3’ | 174 | This paper |
|  | NRF2 R (human) | 5’-CCACTGGTTTCTGACTGGATGT-3’ |  |  |
| 29 | FTH1 F (human) | 5’-CCCCCATTTGTGTGACTTCAT-3’ | 181 | This paper |
|  | FTH1 R (human) | 5’-GCCCGAGGCTTAGCTTTCATT-3’ |  |  |
| 30 | 18S F (mouse) | 5’-CGCCGCTAGAGGTGAAATTCT-3’ | 67 | This paper |
|  | 18S R (mouse) | 5’-CATTCTTGGCAAATGCTTTCG-3’ |  |  |
| 31 | Ccl2 F (mouse) | 5’-TTAAAAACCTGGATCGGAACCAA-3’ | 121 | This paper |
|  | Ccl2 R (mouse) | 5’-GCATTAGCTTCAGATTTACGGGT-3’ |  |  |
| 32 | Ccl5 F (mouse) | 5’-GCTGCTTTGCCTACCTCTCC-3’ | 104 | This paper |
|  | Ccl5 R (mouse) | 5’-TCGAGTGACAAACACGACTGC-3’ |  |  |
| 33 | II12b F (mouse) | 5’-TGGTTTGCCATCGTTTTGCTG-3’ | 123 | This paper |
|  | II12b R (mouse) | 5’-ACAGGTGAGGTTCACTGTTTCT-3’ |  |  |
| 34 | Il1b F (mouse) | 5’-GAAATGCCACCTTTTGACAGTG-3’ | 116 | This paper |
|  | Il1b R (mouse) | 5’-TGGATGCTCTCATCAGGACAG-3’ |  |  |
| 35 | Cxcl5 F (mouse) | 5’-TCCAGCTCGCCATTCATGC-3’ | 117 | This paper |
|  | Cxcl5 R (mouse) | 5’-TTGCGGCTATGACTGAGGAAG-3’ |  |  |
| 36 | Cxcl1 F (mouse) | 5’-CTGGGATTCACCTCAAGAACATC-3’ | 117 | This paper |
|  | Cxcl1 R (mouse) | 5’-CAGGGTCAAGGCAAGCCTC-3’ |  |  |
| 37 | Cxcl16 F (mouse) | 5’-CCTTGTCTCTTGCGTTCTTCC-3’ | 139 | This paper |
|  | Cxcl16 R (mouse) | 5’-TCCAAAGTACCCTGCGGTATC-3’ |  |  |
| 38 | Tnfα F (mouse) | 5’-CAGGCGGTGCCTATGTCTC-3’ | 89 | This paper |
|  | Tnfα R (mouse) | 5’-CGATCACCCCGAAGTTCAGTAG-3’ |  |  |
| 39 | Col3a1 F (mouse) | 5’-CTGTAACATGGAAACTGGGGAAA-3’ | 144 | This paper |
|  | Col3a1 R (mouse) | 5’-CCATAGCTGAACTGAAAACCACC-3’ |  |  |
| 40 | Col4a1 F (mouse) | 5’-CTGGCACAAAAGGGACGAG-3’ | 238 | This paper |
|  | Col4a1 R (mouse) | 5’-ACGTGGCCGAGAATTTCACC-3’ |  |  |
| 41 | Tgfb1 F (mouse) | 5’-CTCCCGTGGCTTCTAGTGC-3’ | 133 | This paper |
|  | Tgfb1 R (mouse) | 5’-GCCTTAGTTTGGACAGGATCTG-3’ |  |  |
| 42 | Ctgf F (mouse) | 5’-GGGCCTCTTCTGCGATTTC-3’ | 151 | This paper |
|  | Ctgf R (mouse) | 5’-ATCCAGGCAAGTGCATTGGTA-3’ |  |  |
| 43 | Col1a1 F (mouse) | 5’-GCTCCTCTTAGGGGCCACT-3’ | 103 | This paper |
|  | Col1a1 R (mouse) | 5’-CCACGTCTCACCATTGGGG-3’ |  |  |
| 44 | Acta2 F (mouse) | 5’-GTCCCAGACATCAGGGAGTAA-3’ | 102 | This paper |
|  | Acta2 R (mouse) | 5’-TCGGATACTTCAGCGTCAGGA-3’ |  |  |
| 45 | Fasn F (mouse) | 5’-GGAGGTGGTGATAGCCGGTAT-3’ | 140 | This paper |
|  | Fasn R (mouse) | 5’-TGGGTAATCCATAGAGCCCAG-3’ |  |  |
| 46 | Srebp1c F (mouse) | 5’-TGACCCGGCTATTCCGTGA-3’ | 61 | This paper |
|  | Srebp1c R (mouse) | 5’-CTGGGCTGAGCAATACAGTTC-3’ |  |  |
| 47 | Scd1 F (mouse) | 5’-TTCTTGCGATACACTCTGGTGC-3’ | 98 | This paper |
|  | Scd1 R (mouse) | 5’-CGGGATTGAATGTTCTTGTCGT-3’ |  |  |
| 48 | Acc F (mouse) | 5’-ATGGGCGGAATGGTCTCTTTC-3’ | 148 | This paper |
|  | Acc R (mouse) | 5’-TGGGGACCTTGTCTTCATCAT-3’ |  |  |
| 49 | Cpt2 F (mouse) | 5’-CAGCACAGCATCGTACCCA-3’ | 172 | This paper |
|  | Cpt2 R (mouse) | 5’-TCCCAATGCCGTTCTCAAAAT-3’ |  |  |
| 50 | Hmgcs2 F (mouse) | 5’-GAAGAGAGCGATGCAGGAAAC-3’ | 166 | This paper |
|  | Hmgcs2 R (mouse) | 5’-GTCCACATATTGGGCTGGAAA-3’ |  |  |
| 51 | Cpt1b F (mouse) | 5’-GCACACCAGGCAGTAGCTTT-3’ | 107 | This paper |
|  | Cpt1b R (mouse) | 5’-CAGGAGTTGATTCCAGACAGGTA-3’ |  |  |
| 52 | Acox1 F (mouse) | 5’-TAACTTCCTCACTCGAAGCCA-3’ | 262 | This paper |
|  | Acox1 R (mouse) | 5’-CTGGGCGTAGGTGCCAATTA-3’ |  |  |
| 53 | Pparα F (mouse) | 5’-TATGGAGTGACATAGAGTGTGCT-3’ | 134 | This paper |
|  | Pparα R (mouse) | 5’-CCACTTCAATCCACCCAGAAAG-3’ |  |  |
|  | | |  |  |
|  |  |  |  |  |
| **FCN3 shRNA sequence** | | | |  |
| NO. | Primers | Sequences | |  |
| 1 | 1#-F | 5’-CCGGGCATCCTGTTACCGATCAAATCTCG AGATTTGATCGGTAACAGGATGCTTTTTG-3’ | |  |
|  | 1#-R | 5’-AATTCAAAAAGCATCCTGTTACCGATCAAAT CTCGAGATTTGATCGGTAACAGGATGC-3’ | |  |
| 2 | 2#-F | 5’-CCGGCTTTAATGGTAACCGTACTTTCTCGA GAAAGTACGGTTACCATTAAAGTTTTTG-3’ | |  |
|  | 2#-R | 5’-AATTCAAAAACTTTAATGGTAACCGTACTTT CTCGAGAAAGTACGGTTACCATTAAAG-3’ | |  |
