# Supplementary material for: Ficolin 3 promotes ferroptosis in HCC by downregulating IR/SREBP axis-mediated MUFA synthesis
Source: J Exp Clin Cancer Res. 2024 May 3;43:133. doi: 10.1186/s13046-024-03047-2 (PMC11067213; doi:10.1186/s13046-024-03047-2)
Supplement: Supplementary file 5 — Supplementary Material 5 [file 13046_2024_3047_MOESM5_ESM.docx]

**Supplemental figures and figure legends**


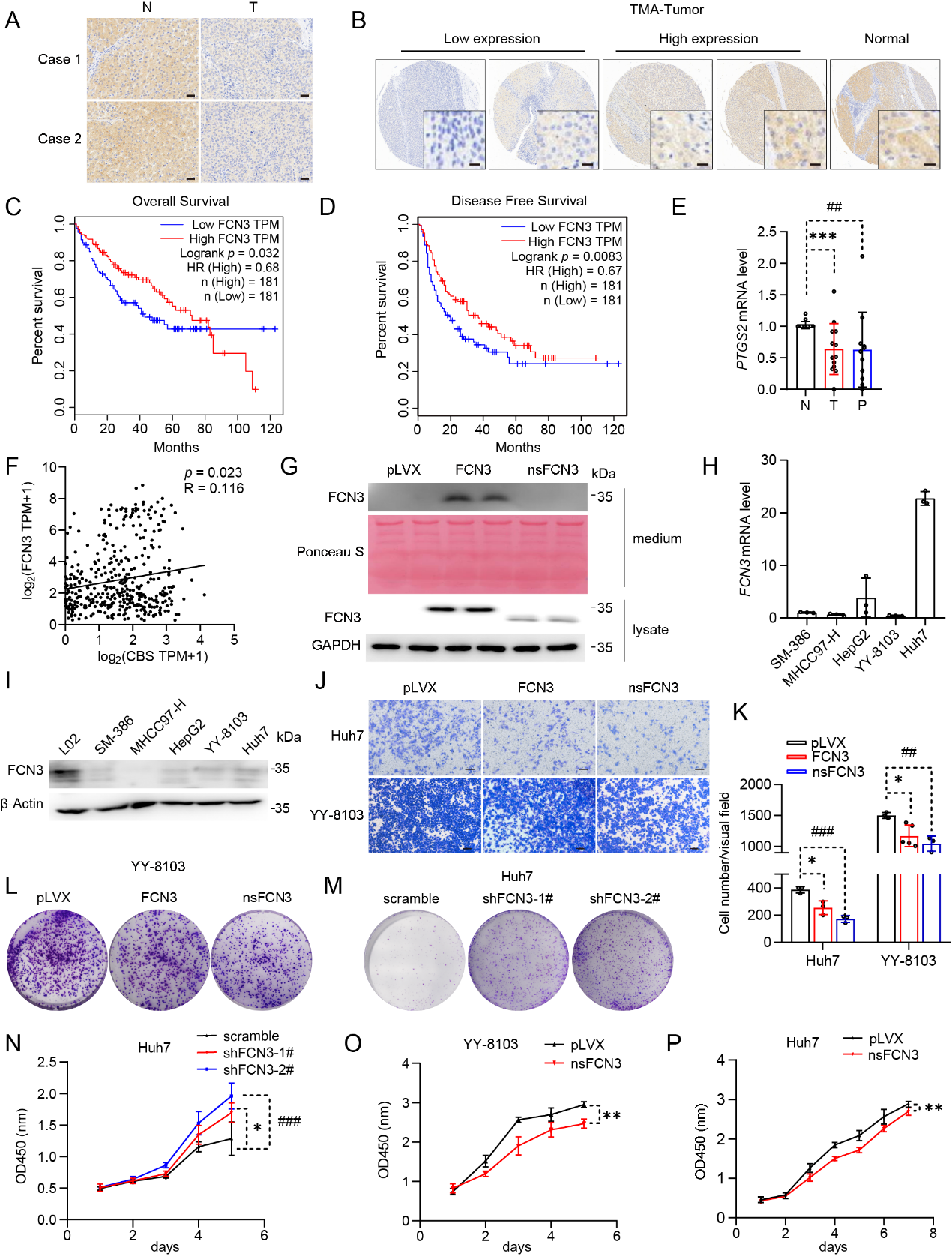


**Fig. S1. FCN3 inhibits the migration and growth of HCC cells through its intracellular functionality**

(A) Representative images of FCN3 IHC staining in HCC tumor and paired adjacent nontumor tissues. Scale bar, 40 μm. (B) Representative images of FCN3 IHC staining for different H-score level in TMA slides. Scale bar, 20 μm. (C-D) Kalpan–Meier analysis of overall survival (C) and disease-free survival (D) data containing 362 patients from TCGA database. (E) mRNA expression of *PTGS2* in HCC (T), PVTT (P) and paired adjacent nontumor (N) tissues. (F) Correlation analysis of *CBS* and *FCN3* expressions based on TCGA database. (G) Immunoblot of FCN3 in YY-8103 cells overexpressing FCN3, nsFCN3 or control. GAPDH was used as a loading control. (H) mRNA level of *FCN3* in different HCC cell lines. (I) Immunoblot of FCN3 in L02 and different HCC cell lines. β-Actin was used as a loading control. (J) Representative images of migrating Huh7 and YY-8103 cells that cultured in trans-well plates. Scale bar, 100 μm. (K) Quantification of the average number of migrating Huh7 and YY-8103 cells in (J). (L) Representative images of the crystal violet assay in YY-8103 cells. (M) Representative images of the crystal violet assay in FCN3-knockdown Huh7 cells. (N) CCK8 assay in FCN3-knockdown Huh7 cells. (O-P) CCK8 assay in nsFCN3-overexpressed YY-8103 (O) and Huh7 (P) cells.

Data are from one representative experiment of three independent experiments (J-M). Data are presented as mean ± SD. Significance was assessed by Mann-Whitney *U* test (E), Spearman correlation (F), Student’s *t* test (K). *^, #^*p* < 0.05, **^, ##^*p* < 0.01, ***^, ###^*p* < 0.001 compared with the control group.


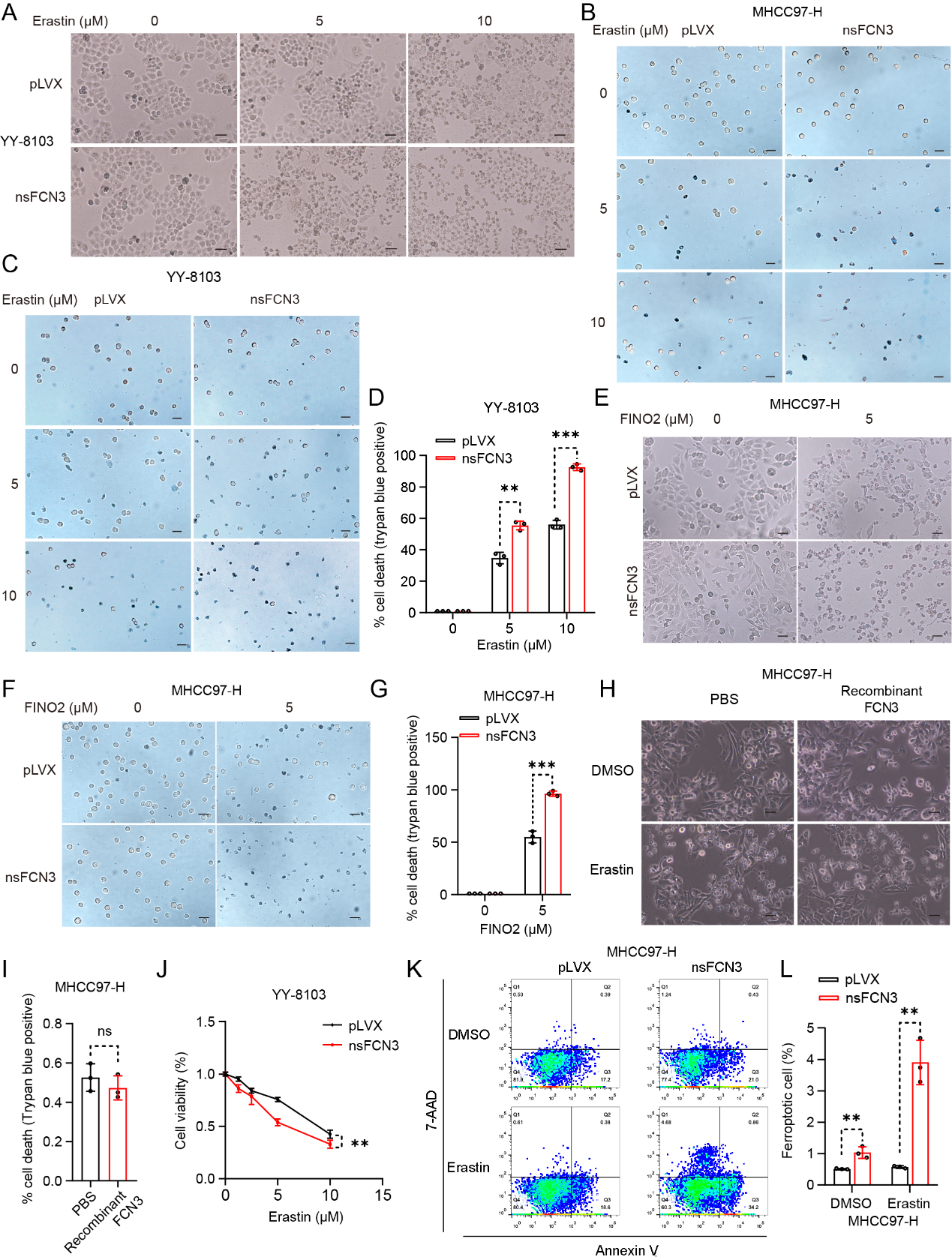


**Fig. S2. nsFCN3 overexpression heightens the susceptibility of HCC cells to ferroptosis**

(A) Representative images of nsFCN3-overexpressed YY-8103 cells treated with erastin. Scale bar, 100 μm. (B-C) Representative images of trypan blue staining for nsFCN3-overexpressed MHCC97-H (B) and YY-8103 (C) cells treated with erastin. Scale bar, 100 μm. (D) Quantification of trypan blue staining for death cell in (C). (E) Representative images for nsFCN3-overexpressed and control MHCC97-H cells treated with FINO2 for 24 h. Scale bar, 100 μm. (F-G) Representative images (F) and quantification (G) of trypan blue staining for nsFCN3-overexpressed and control MHCC97-H cells treated with FINO2. Scale bar, 100 μm. (H) Representative images of MHCC97-H cells treated with 300 ng/mL FCN3 recombinant protein, followed by treatment with erastin. Scale bar, 100 μm. (I) Quantification of trypan blue staining for death cell in (H). (J) Viability of nsFCN3-overexpressed and control YY-8103 cells treated with indicated concentration of erastin for 24 h. (K) Representative images of ferroptosis cells analyzed by flow cytometry for nsFCN3-overexpressed and control MHCC97-H cells. (L) Proportions of ferroptosis cells in (K).

Data are from one representative experiment of three independent experiments (A-J). Data are presented as mean ± SD. Significance was assessed by Student’s *t* test (D, G, I, L). ***p* < 0.01, ****p* < 0.001 compared with the control group. ns, not significant.


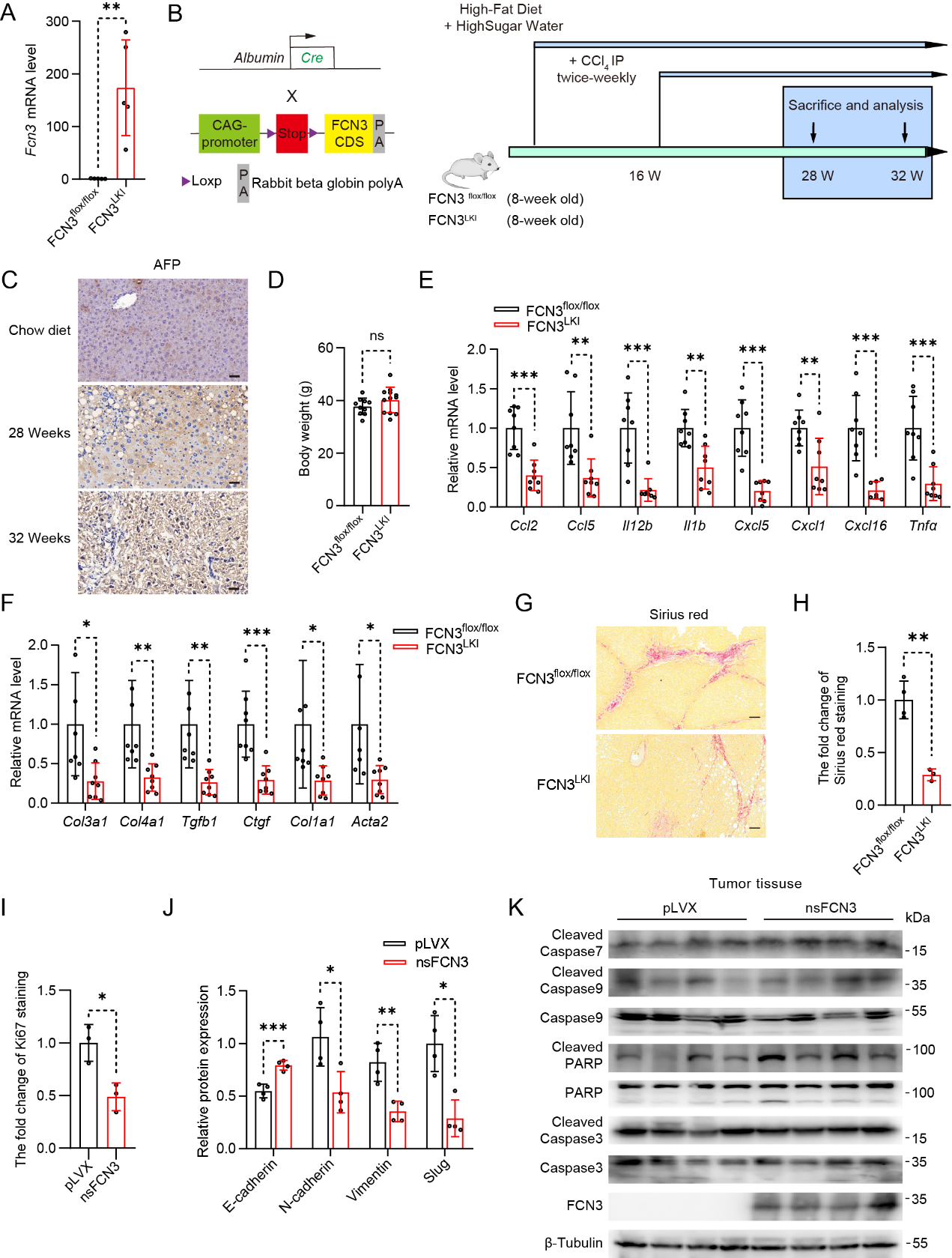


**Fig. S3. The role of FCN3 in primary HCC and xenograft tumor**

(A) mRNA level of *Fcn3* in the liver of FCN3^LKI^ and control mice. (B) Schematic diagram of HCC model. (C) Representative images of AFP IHC staining in the HCC section of FCN3^LKI^ mice in figure 3D. Scale bar, 40 μm. (D) Body weights of mice at week 32. (E) mRNA levels of inflammatory genes in the liver tissues of FCN3^LKI^ and control mice at week 32. (F) mRNA levels of fibrosis-related genes in the liver tissues of FCN3^LKI^ and control mice at week 32. (G) Representative images of Sirius red staining in mouse liver at week 32. Scale bar, 40 μm. (H) Quantification of Sirius red staining in (G). (I) Quantification of Ki67 staining in figure 3N. (J) Quantification of EMT-related proteins figure 3O. (K) Immunoblots of apoptosis-related proteins in xenograft tumors in figure 3H.

Data are presented as mean ± SD. Significance was assessed by Student’s *t* test (A, D, F, H, I), Mann-Whitney *U* test (E, J). **p* < 0.05, ***p* < 0.01, ****p* < 0.001 compared with the control group. ns, not significant.


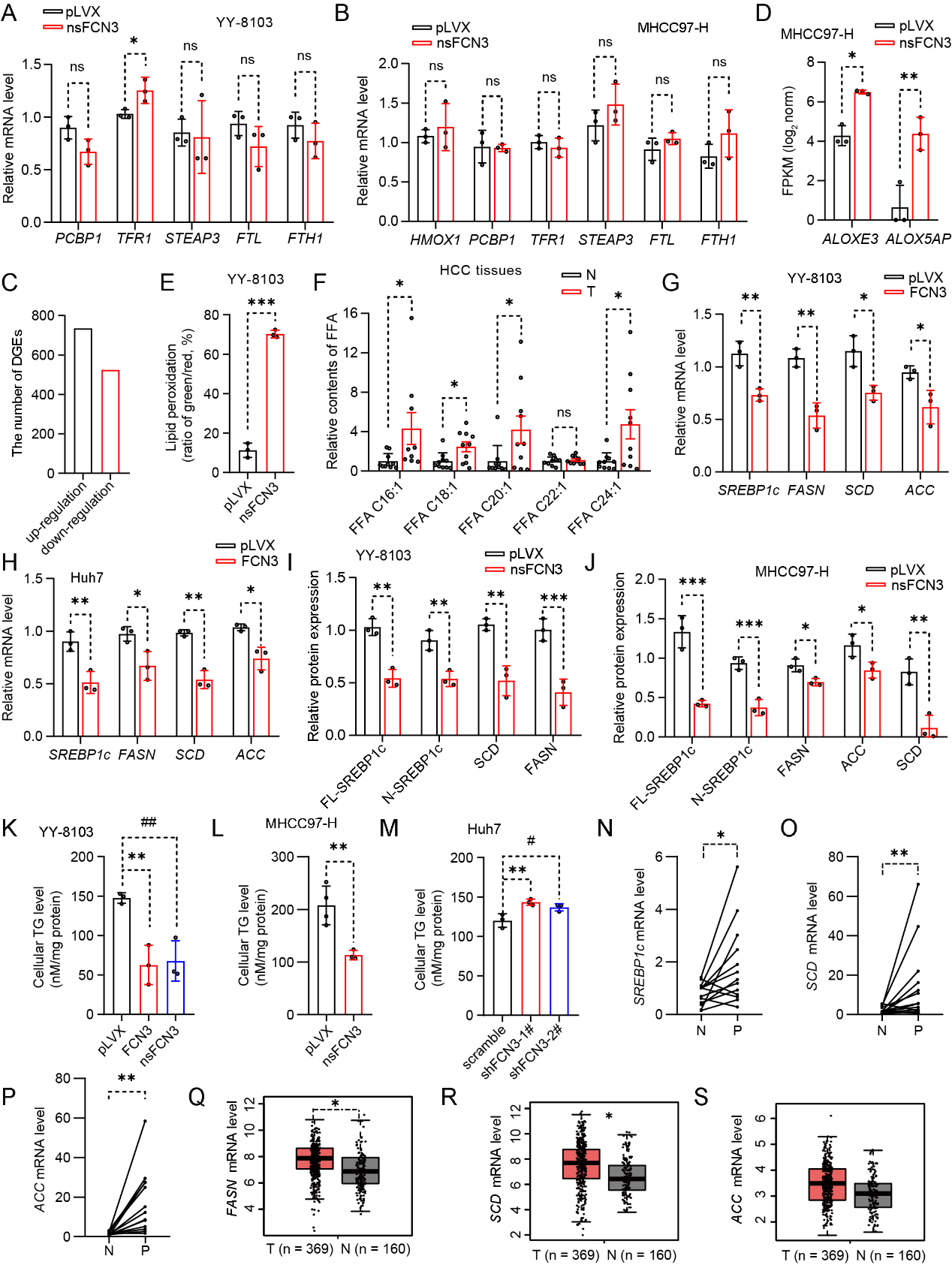


**Fig. S4. FCN3 overexpression inhibits expression of SREBP1c and lipid synthases**

(A-B) mRNA levels of iron metabolism-related genes in nsFCN3-overexpressed YY-8103 (A) and MHCC97-H (B) cells. (C) The number of DEGs in nsFCN3-overexpressed and control MHCC97-H cells analyzed with RNA-seq (*n* = 3 per group). (D) mRNA levels of ferroptosis-related genes based on RNA-seq result. (E) Quantification of BODIPY-C11 stained nsFCN3-overexpressed YY-8103 cells treated with 10 μM erastin for 12 h in figure 4I. (F) Quantification of MUFAs in tumor tissues (T) and paired normal adjacent tissue (N) in figure 4L. (G-H) mRNA levels of lipogenic genes in FCN3-overexpressed YY-8103 (G) and Huh7 (H) cells. (I) Quantification of lipogenic proteins in nsFCN3-overexpressed YY-8103 cells in figure 5E. (J) Quantification of lipogenic proteins in nsFCN3-overexpressed MHCC97-H cells in figure 5F. (K) The content of TG in FCN3- or nsFCN3-overexpressed YY-8103 cells treated with 100 μM palmitic acid (PA) (n = 3). (L) The content of TG in nsFCN3-overexpressed MHCC97-H cells treated with 100 μM PA (n = 3). (M) The content of TG in FCN3-knockdown Huh7 cells treated with 100 μM PA (*n* = 3). (N-P) mRNA level of *SREBP1c* (N), *SCD* (O) and *ACC* (P) in 15 paired adjacent and PVTT tissues. (Q-S) mRNA levels of *FASN* (Q), *SCD* (R), and *ACC* (S) in normal human liver tissues (N) and HCC tissues (T) in published data from TCGA database.

Data are from one representative experiment of three independent experiments (G-J). Data are presented as mean ± SD. Significance was assessed by unpaired Student’s *t* test (A, B, E, G, H, I, J, K, L), Mann-Whitney *U* test (D, F, Q, R, S), one-way ANOVA (M), paired Student’s *t* test (N, P), Wilcoxon matched-pairs signed rank test (O). *^, #^*p* < 0.05, **^, ##^*p* < 0.01, ****p* < 0.001 compared with the control group. ns, not significant.


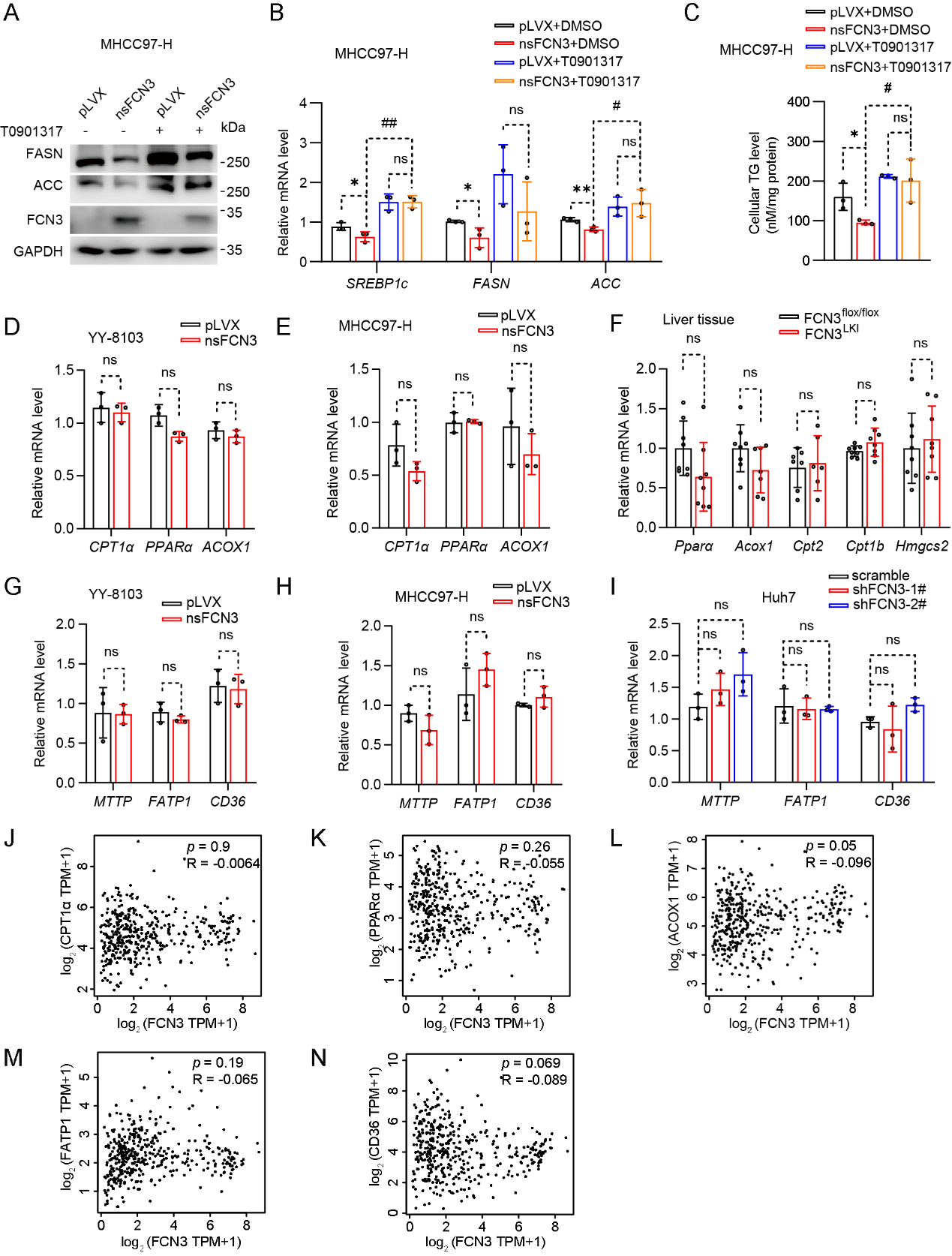


**Fig. S5. FCN3 exerts minimal effects on the expression of genes related to FAO or lipid transport**

(A) Immunoblots of DNL and EMT related proteins in nsFCN3-overexpressed MHCC97-H cells treated with T0901317 (10 µM) for 24 h. GAPDH was used as a loading control. The experiment was repeated twice. (B) mRNA levels of lipogenic genes in nsFCN3-overexpressed and control MHCC97-H cells treated with T0901317 (10 µM) for 24 h. (C) TG contents in nsFCN3-overexpressed MHCC97-H cells treated with T0901317 (10 µM) and PA (100 µM). (D-E) mRNA levels of FAO genes in nsFCN3-overexpressed YY-8103 (D) and MHCC97-H (E) cells. (F) mRNA levels of FAO genes in the liver tissues of FCN3^LKI^ and control mice. (G-H) mRNA levels of lipid transport genes in nsFCN3-overexpressed YY-8103 (G) and MHCC97-H (H) cells. (I) mRNA levels of lipid transport genes in FCN3-knockdown Huh7 cells. (J-L) Correlation analysis of *CPT1α* (J), *PPARα* (K), *ACOX1* (L) and *FCN3* expressions based on TCGA database. (M-N) Correlation analysis of *FATP1* (M), *CD36* (N) and *FCN3* expressions based on TCGA database.

Data are presented as mean ± SD. Significance was assessed by Student’s *t* test (B, C, D, E, F, G, H), one-way ANOVA (I). *^, #^*p* < 0.05, **^, ##^*p* < 0.01 compared with the control group. ns, not significant.


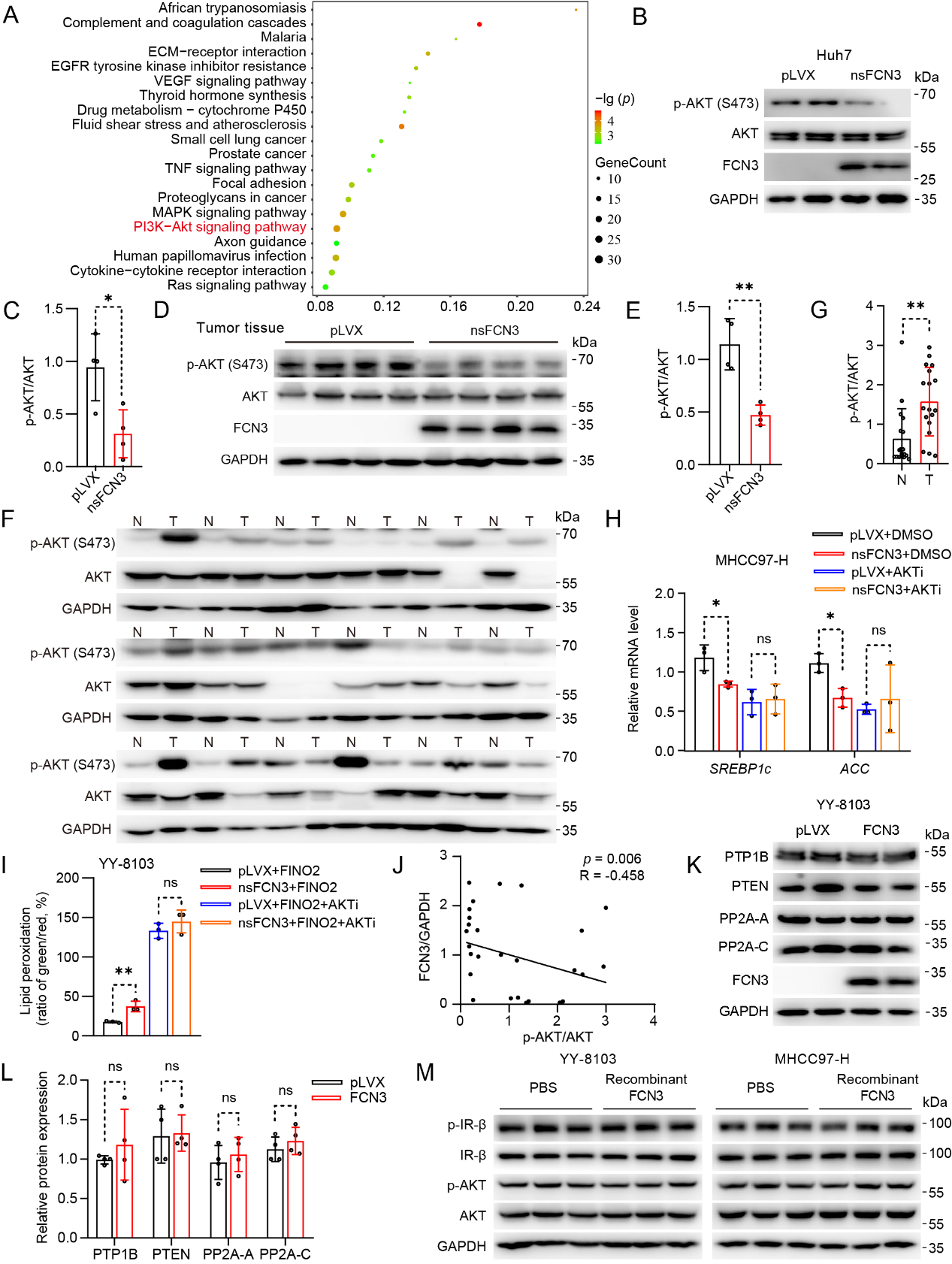


**Fig. S6. FCN3 inhibits the IR/AKT signaling pathway**

(A) The KEGG pathway enrichment based on the DEGs (|log_2_ Fold Change| > 1, *p* value < 0.05) in nsFCN3-overexpressed and control MHCC97-H cells analyzed with RNA-seq (*n* = 3). (B-C) Immunoblot (B) and quantification (C) of p-AKT in nsFCN3-overexpressed Huh7 cells. GAPDH was used as a loading control. (D-E) Immunoblot (D) and quantification (E) of p-AKT in nsFCN3-overexpressed and control tumors. GAPDH was used as a loading control. (F) Immunoblot of p-AKT in 18 pairs of HCC (T) and adjacent nontumor (N) tissues. GAPDH was used as a loading control. (G) Quantitative analysis of p-AKT/AKT ratio from (F). (H) mRNA levels of lipogenic genes in nsFCN3-overexpressed MHCC97-H cells treated with AKTi for 24 h, AKTi: 10 μM. (I) Quantification of BODIPY-C11 stained nsFCN3-overexpressed YY-8103 cells treated with FINO2 and AKTi in figure 6H. (J) Correlation analysis of p-AKT/AKT ratio and FCN3/GAPDH ratio quantitated in figure 1E and panel G. (K-L) Immunoblots (K) and quantification (L) of indicated proteins in FCN3-overexpressed YY-8103 cells. GAPDH was used as a loading control. (M) Immunoblot of p-IR-β and p-AKT in MHCC97-H and YY-8103 cells after treated with 300 ng/mL FCN3 recombinant protein. GAPDH was used as a loading control.

Data are presented as mean ± SD. Significance was assessed by Student’s *t* test (C, E, H, L), Mann-Whitney *U* test (G, I), Spearman correlation (J). **p* < 0.05, ***p* < 0.01, ****p* < 0.001 compared with the control group. ns, not significant.
